# Exploration adjustment by ant colonies

**DOI:** 10.1098/rsos.150533

**Published:** 2016-01-27

**Authors:** Carolina Doran, Martin C. Stumpe, Ana Sendova-Franks, Nigel R. Franks

**Affiliations:** 1 School of Biological Sciences, Bristol Life Sciences Building, 24 Tyndall Avenue, Bristol BS8 1TQ, UK; 2 Champalimaud Neuroscience Programme, Champalimaud Centre for the Unknown, Avenida Brasília, Lisbon 1400-038, Portugal; 3 AnTracks Computer Vision Systems, Mountain View, CA, USA; 4 Department of Engineering Design and Mathematics, UWE Bristol, Coldharbour Lane, Bristol BS16 1QY, UK

**Keywords:** exploration, *Temnothorax albipennis*, division of labour, flexibility

## Abstract

How do animals in groups organize their work? Division of labour, i.e. the process by which individuals within a group choose which tasks to perform, has been extensively studied in social insects. Variability among individuals within a colony seems to underpin both the decision over which tasks to perform and the amount of effort to invest in a task. Studies have focused mainly on discrete tasks, i.e. tasks with a recognizable end. Here, we study the distribution of effort in nest seeking, in the absence of new nest sites. Hence, this task is open-ended and individuals have to decide when to stop searching, even though the task has not been completed. We show that collective search effort declines when colonies inhabit better homes, as a consequence of a reduction in the number of bouts (exploratory events). Furthermore, we show an increase in bout exploration time and a decrease in bout instantaneous speed for colonies inhabiting better homes. The effect of treatment on bout effort is very small; however, we suggest that the organization of work performed within nest searching is achieved both by a process of self-selection of the most hard-working ants and individual effort adjustment.

## 1. Background

Every day we see examples of groups of individuals behaving collectively and creating patterns we would not be able to predict from the behaviour of the individuals alone; from action potential patterns in populations of neurons all the way to task allocation in animal groups [1–3]. The search for the rules underlying these complex phenomena is a major growth point within the biological sciences.

Social insects have to perform several different tasks in order to maintain their nests and keep the group safe and fed, such as caring for their brood, patrolling the nest, foraging for food and scouting for new homes. However, there is no central individual instructing the others what to do, and so, the process of allocating tasks is said to be self-organized, whereby individuals responding to local cues achieve a functional global structure [4–6].

In an unpredictable environment, flexibility in task allocation is crucial, not only in terms of which tasks should be performed, but also with regard to how much effort should be put into each task. Much research has focused on understanding how social insect colonies organize their work [4,5,7]. Division of labour has often been seen as the primary reason for the success of social insects [8–10]. Long ago, Adam Smith [11] studied how groups in human societies could increase the efficiency of their work by dividing labour among the different individuals. In other biological systems, such as social insects, the term ‘division of labour’ is used when groups of individuals are co-adapted through divergent specialization to increase their overall inclusive fitness [5]. Furthermore, it has been proposed that even in colonies with monomorphic workers, i.e. colonies in which there is no physical diversity among specialized worker castes, certain individuals are more prone to perform certain tasks and hence become specialists for such tasks [12]. There are several models of how this organization could be achieved. The ‘foraging-for-work’ model suggests that there is a spatial distribution of tasks and individuals move between them according to task demand, i.e. discrepancies between the local task force and the work needed in that particular task [13]. Other models argue that social interaction among members of the colony is the key feature for successful division of labour by which individuals choose which task to perform depending on how many individuals are engaged in other tasks [14,15]. Currently, one of the main models for division of labour in social insects assumes that individuals will perform a task whenever their internal threshold for that specific task has been crossed [6]. This model assumes individuals vary in their response thresholds and hence different individuals perform different tasks [6,16]. All of these mechanisms might contribute simultaneously to the process of task allocation and possibly to the understanding of how work is organized in general. In line with the ‘foraging-for-work’ strategy, there might also be a spatial distribution of the amount of work to be done within a single task; social interactions might also play a role in transferring information regarding how much work is left to be done; and finally, individuals probably also vary in their response threshold within each task, hence the study of division of labour might shed some light onto the process of work organization within a single task.

Exploration is a very important task as it allows animals to discover resources and detect the presence of potential competitors [17]. Ant colonies provide some of the richest examples for the study of collective phenomena such as collective exploration. They inhabit an enormous variety of environments with different species searching for different types of resource under various predation risks [18,19]. There are ant species that search on the ground, underground, in trees and recent research has shown they are able to respond even to environments with microgravity, though very poorly as they are not as effective there, in their collective search [8,20].

*Temnothorax albipennis* ant colonies explore on the ground and their search for potential nest sites is well studied [3,21–23]. These colonies generally prefer cavities with small entrances (so that they can defend the nest effectively), cavities with a specific height (so that they can tend their brood items) and finally cavities that are dark (which in the wild is likely to be a good indicator of how exposed the nest is to the outside world) [21]. Scouting effort at the colony level appears to be finely tuned to the value of the current nest site [24–26]. These colonies adjust how fast they move to a new nest depending on the value difference between current and target nest and this collective adjustment is actually driven by individuals recruiting faster when faced with a more profitable move, i.e. when the difference in value between current and target nest is greater [27].

Task allocation is highly dynamic, in that the number of individuals performing a specific task is constantly changing depending on the colony’s need for that task [15,24]. Several factors seem to play a role in which tasks an individual will perform, namely age, spatial location, experience and corpulence; however, the latter seems to be the one with the greatest influence in certain tasks in *T. albipennis* ants [28]. Arguably, any ant able to perceive a stimulus should be able to respond appropriately [29]; however, the intensity with which this ant responds might still vary and depend on its internal response threshold. Division of labour has been extensively studied particularly in social insects, with few studies investigating how the amount of work within the same task is organized [30]. Furthermore, the majority of such studies focus on tasks that are discrete, i.e. having a recognizable beginning and an end, as is the case of bumblebees fanning in order to cool down their nest or ants carrying brood items to the new colony’s nestsite [30,31]. These tasks naturally end when the nest is sufficiently cool or when there are no more brood items to be transported. In tasks such as nest searching where the outcome is highly unpredictable, the task is often open-ended as the goal might never be achieved, hence individuals must decide when to stop searching before the task is completed.

This study focuses on the distribution of work within a single task, scouting in the absence of a target nest. We therefore specifically investigate how the collective search pattern observed for colonies that inhabit nests of different value is affected both by the total amount of scouting and also by the effort invested in each exploratory bout.

## 2. Material and methods

### 2.1 Colonies

Ten colonies were collected from the coast of Dorset, UK; five in February and five in June 2012. Colony size ranged between 63 and 195 workers with a similar number of brood and all colonies had a queen. Colonies were cultured and fed according to established procedures [9].

### 2.2 Experiment

**Figure 1.**
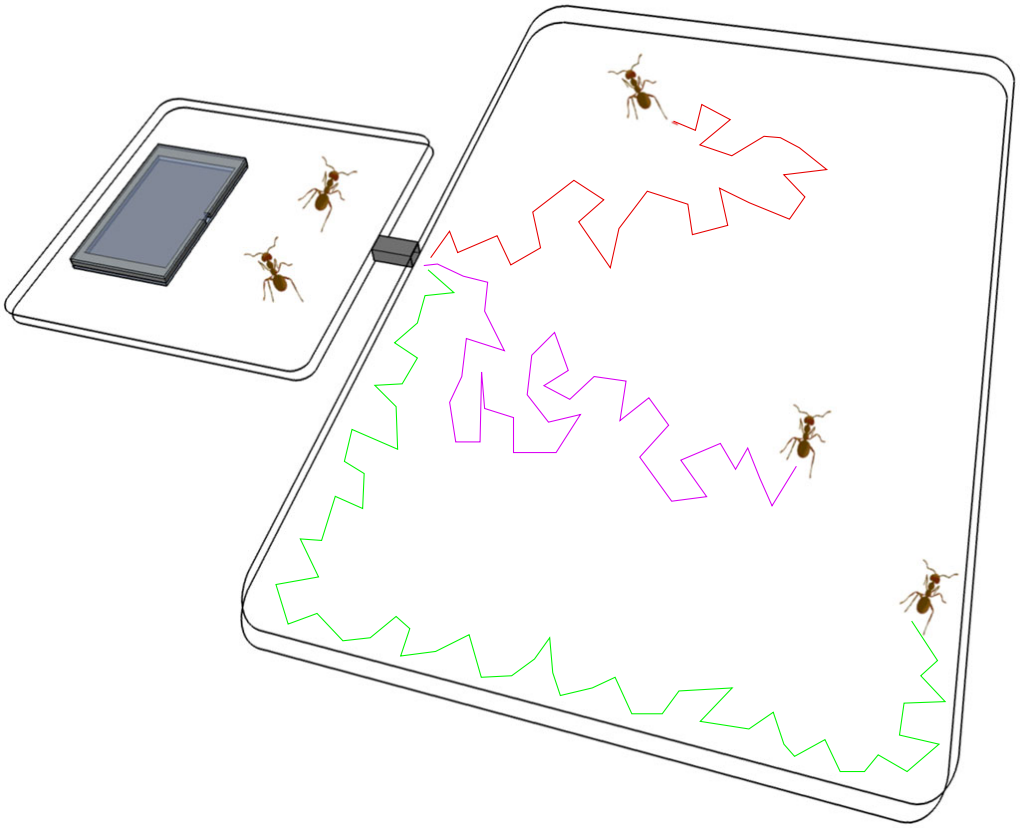
Experimental set-up: square Petri dish (10 × 10 cm) connected via a plastic tunnel (4 cm) to a rectangular scouting arena (28 × 37 cm). A video camera was placed to film the entire scouting arena. This set-up did not contain a new target nest site: the scouting arena was empty. The coloured lines show three different trajectories performed by individual ants as a representation of the ones recorded by the software AnTracks.

We allowed each of the 10 colonies sequentially to inhabit five different nests of different value, namely Poor (light roof, low ceiling and large entrance), Satisfactory (light roof, low ceiling and medium entrance size), Medium (light roof, low ceiling and narrow entrance), Good (light roof, high ceiling and narrow entrance) and Excellent (dark roof, high ceiling and narrow entrance). All nests consisted of two glass slides sandwiching a cardboard perimeter all with a cavity size of 35 × 60 mm; low ceilings are 1 mm and high ceilings are 2 mm; wide entrances are 60 mm, medium size entrances 4 mm and narrow entrances 1 mm (nest designs were taken from [25]). We placed a video camera above the scouting arena and filmed it for 270 min. Every colony experienced each one of the five nesting conditions according to a Latin square design. Treatments were separated by a period of one week in order to allow time for colonies to settle into their nests and avoid improvement based on experience [32]. Colonies inhabited each test nest for a period of 7 days prior to experiments. On the 8th day, we placed the nest containing the focal colony in the experimental arena and allowed individuals that exited their nests onto an open white rectangular environment (figure 1).

### 2.3 Data analysis

We analysed all the videos of the scouting arena with AnTracks (www.antracks.org), software which records the position of every ant in each frame in the scouting arena, though it cannot determine if the same individual enters the scouting arena several times. We then used this information to calculate a set of different measures of scouting effort, as follows:

(1) Number of bouts: number of times any ant was recorded entering the scouting arena for every colony in each of the five different nest qualities.
(2) Bout path length: overall distance of each bout. Total path length is the sum of all the bout path lengths in each colony.
(3) Bout duration: time between the ant entering and leaving the arena. The total exploration duration is the sum of all the bout durations in each colony.
(4) Bout instantaneous speed: median distance covered per second in each bout.

We used Python for calculating the parameters mentioned and details can be found in the electronic supplementary material, section 2a,b.

### 2.4 Statistical analysis

We analysed the effect of current nest value (predictor factor) on the total number of bouts (response variable) with a generalized linear mixed model for a Poisson response with a log link using the glmer function of the lme4 package with lmerTest for *p*-value estimation in R (v. 3.1.2) [33]. We also analysed the effect of current housing conditions on the amount of work colonies accomplished when scouting with a linear mixed model, using the lmer function in the lme4 package, again with lmerTest for *p*-value estimation in R (v. 3.1.2). The response variable was either the total path length or the total exploration duration (that is the sum of all bout path lengths or bout durations, respectively), both square-rooted (electronic supplementary material, section d,e, and figures S6 and S7). To analyse how the work performed within a bout changed according to the different nesting conditions, we again ran a linear mixed model using the same function, lmer. The response variable was log of bout path length, log of bout duration or bout instantaneous speed (electronic supplementary material, section g–i, and figures S17–S19). For all models, the predictors were current nest value (a fixed factor) and colony (a random factor varying around the intercept). We ran these models with polynomial and treatment contrast to test for significance of linear, quadratic, cubic and fourth-order trends and differences between the different nest values (pairwise comparison). Pairwise comparisons were adjusted using Bonferroni corrections.

## 3. Results

### 3.1 Number of bouts

**Figure 2.**
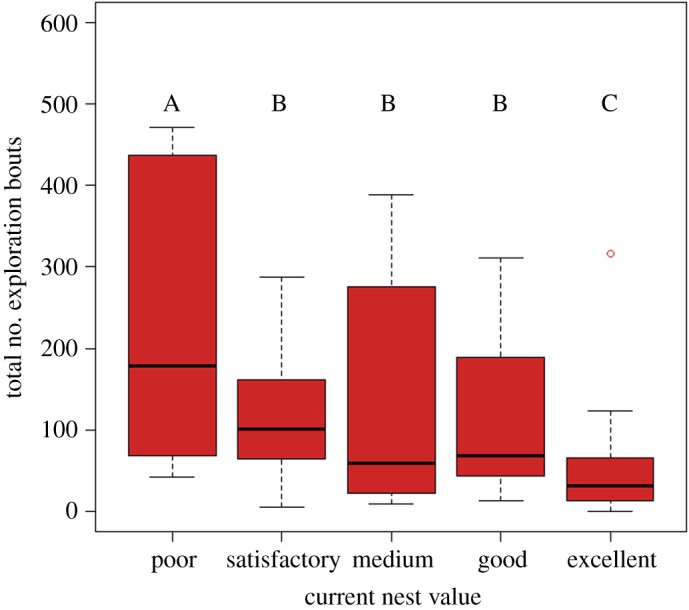
Boxplots of the total number of exploration bouts for colonies inhabiting each of the five different nest qualities. Black lines indicate medians, the boxes delineate the interquartile ranges, the whiskers represent the nearest value within 1.5 of the interquartile range and capital letters denote the significant differences between the different nests after Bonferroni corrections.

The total number of bouts decreased with increasing current nest value (table 1 and figure 2). Both Poor and Excellent current nests were significantly different from each other and from all other nest values. The polynomial contrast was significant for both a linear and cubic trend, and the overall trend was downward, i.e. an increase in current nest value tended to decrease the number of bouts (electronic supplementary material, figure S1). The residuals were normally distributed (Shapiro–Wilk normality test: *W*=0.987, *p*=0.8548, electronic supplementary material, figures S2–S4) indicating that the model was a good fit with colonies deviating significantly from the overall intercept and hence justifying inclusion of the random factor (electronic supplementary material, figure S5). This means that, in accord with previous data [24,25], colonies put more effort into looking for a new home when inhabiting nests of lower value. The R code and output used in this analysis can be found in the electronic supplementary material, section c.

**Table 1.** Summary of all the statistical results: *p*-values for the treatment effect and the polynomial contrasts for the five treatment levels for all six models; the predictor fixed factor was always the treatment (five levels of nest value); the responses are indicated in the column labels; *p*-values significant at the 5% significance level are in italic. The number of observations was 50 and 6491 for colony-level and individual-level measurements, respectively.

	colony level			individual level		
	no. bouts	total path length	total exploration duration	bout path length	bout duration	bout instantaneous speed
treatment effect						
nest value	<*2*×*10*^−*16*^	*6.13*×*10*^−*6*^	*2.884*×*10*^−*3*^	*0.0296*	*9.651*×*10*^−*9*^	<*2*×*10*^−*16*^
polynomial contrasts						
linear	<*2*×*10*^−*16*^	*1.82*×*10*^−*5*^	*3.21*×*10*^−*4*^	*0.0108*	*1.13*×*10*^−*7*^	<*2*×*10*^−*16*^
quadratic	0.497	0.95391	0.740538	0.7178	0.392	0.140
cubic	<*2*×*10*^−*16*^	*0.00684*	0.051	0.3540	0.527	*4.82*×*10*^−*8*^
fourth order	0.419	0.47896	0.7	0.3180	0.134	0.176

### 3.2 Total amount of work

**Figure 3.**
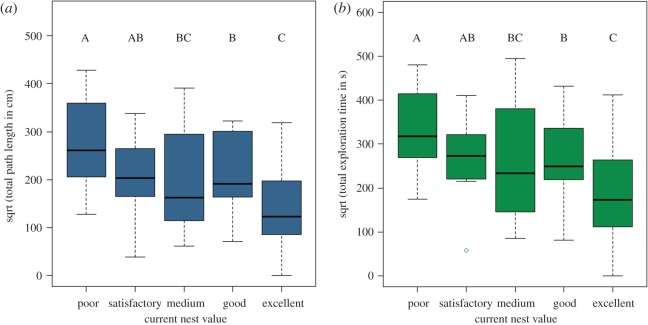
Boxplots of the different measurements of collective effort for the different housing conditions: (*a*) square root of total path length; (*b*) square root of total exploration time. For both cases, black lines indicate the medians, the boxes delineate the interquartile ranges, the whiskers represent the nearest value within 1.5 of the interquartile range and capital letters denote the significant differences between the different housing conditions after Bonferroni corrections.

Colonies explored less when living in better housing conditions. We measured collective work by summing the path lengths and all the durations of all bouts for each colony for every one of the different housing conditions (when the number of bouts was zero, we considered both the total path length and duration also to be zero). For both cases, adding current nest value to the model showed a significant effect (table 1). Both the total path length explored and total exploration duration decreased linearly with the increase in current nest value (table 1 and figure 3). As for the number of bouts, the *p*-value for total path length was significant not only for the linear trend but also for the cubic trend. The same was true for total exploration time though the cubic trend there was marginally non-significant (table 1). Again, the overall trend was downward, meaning that both total path length and total exploration time decreased as current nest value increased (electronic supplementary material, figure S8). For both cases, pairwise contrasts show that Satisfactory, Medium and Good current nests are not significantly different from each other. Good nests are significantly different from Poor current nests, and Excellent current nests are significantly different from all the other nest qualities except Medium. The residuals were not significantly different from normality as shown by Shapiro–Wilk normality tests (total path length: *W*=0.9791, *p*=0.52979; total exploration time: *W*=0.9819, *p*=0.6329, electronic supplementary material, figures S9–S13) indicating that both models are a good fit. Furthermore, colonies deviated significantly from the overall intercept and hence inclusion of the random factor was necessary (electronic supplementary material, figures S12 and S16). Total path length and total exploration duration divided by total number of bouts showed a significant positive linear trend with increasing nest value (electronic supplementary material, figure S33). The R code and output used in this analysis can be found in the electronic supplementary material, section d,f,k.

### 3.3 Distribution of work within bouts

**Figure 4.**
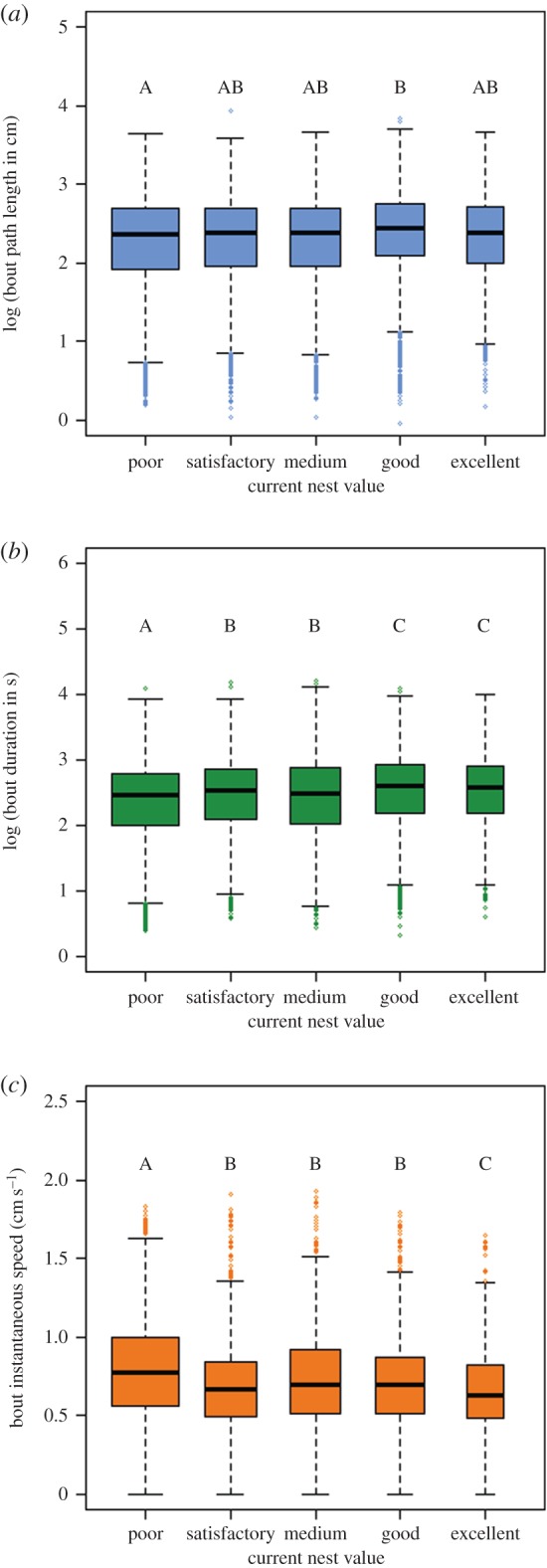
Boxplots of the different measurements for the distribution of work: (*a*) log of bout path length; (*b*) log of bout duration; (*c*) bout instantaneous speed. For all cases, black lines indicate the medians, the boxes delineate the interquartile ranges, the whiskers represent the nearest value within 1.5 of the interquartile range and capital letters denote the significant differences between the different housing conditions after Bonferroni corrections.

Even though the total path length and total exploration time decreased significantly with better housing conditions, the path length and duration of individual bouts showed a significant trend in the opposite direction. For both cases, adding current nest value to the model showed a significant effect (table 1). Furthermore, the path length covered in each bout increased with an increase in current nest value, and so did bout duration (table 1 and figure 4*a*,*b*). Finally, the instantaneous speed of ants decreased during scouting, when their current nest was of higher value. For all these parameters of bout exploration effort, the effect size was very small, with path length being the weakest, as shown by the pairwise contrast results (figure 4*a*). The only significant differences observed were between Poor and Good current nests. The addition of nest value to the model had a significant effect with instantaneous speed decreasing with current nest value increase (table 1 and figure 4*c*). Both the linear trend and the cubic trend were good fits. Furthermore, the overall trend was downward (electronic supplementary material, figure S20). For all three measurements, the residuals only deviate from normality marginally (electronic supplementary material, figures S21–S32) indicating a good model fit. Colonies deviated significantly from the overall intercept and hence inclusion of the random factor colony was necessary (electronic supplementary material, figures S24, S28 and S32).

## 4. Discussion

We manipulated the environment of *T. albipennis* ant colonies by housing them in nests of different value and measured how this influenced both the collective effort and the effort performed in each exploratory bout. We suggest that both the amount of scouting and the flexible behaviour of individuals underpin the changes observed in the collective search pattern.

In accordance with several other studies, here colonies also put a smaller collective effort into exploring their environment when their current housing conditions are of high value [24–26]. The number of bouts, the total path length and total exploration duration significantly decrease with an increase in current nest value. But how is this collective pattern obtained? Is this reduction in the collective search effort just a result of a decrease in the number of active scouts? Or are individuals actually adjusting their search behaviour in accordance with their current housing conditions?

Even though both the total path length and total exploration time decreased significantly in better housing conditions, the path length and duration of individual bouts showed a trend in the opposite direction: bout path length and bout duration increased significantly with current nest value, though the effect was rather weak for bout path length. In addition, the instantaneous speed within individual bouts showed a significant decrease when housing conditions were better. In sum, this means that individuals are exploring for longer periods as a consequence of moving at a slower pace and not necessarily covering more ground. However, the effect size for these parameters was very small. Arguably, as housing conditions gradually improve, only few very active individuals remain scouting. Hence, the increase in path length and duration observed in each bout might be a result of the presence of only the most hard-working scouts actively searching. However, we would then expect a gradual decrease in the variance with nest value increase, which is not the case. Furthermore, dividing both the total path length and the total exploration by the number of bouts does not lead to a loss of any trend; instead we have an increase in response with increasing nest value, hence the decline in the total amount of work cannot be explained solely by the decline in the total amount of scouting.

Previous research has shown how a small number of individuals can have a significant effect on the collective. For this same species, only a few ants could be responsible for distributing food for the entire colony as well as responsible for blocking big entrances with sand, and increasing nest safety [34,35]. Hence, in this study, there might be a combination of two processes: a self-selecting process such that only the most hard-working scouts are active as current nest value increases, and a process whereby such hard-working individuals adjust their search effort according to current housing conditions. In *Linepithema humile* ants, it has been shown that exploring individuals adjust the shape of their paths in response to the number of ants in the arena [36]. Here, we show that colonies organize the amount of work for the same task adjusting not only the size of the workforce but also the amount of effort invested while scouting. When housing conditions are good, the need for a new home is reduced. Hence individuals might decide, before leaving their home, that it is worth prolonging their search in order to be more selective and compensate for the decrease in the number of active scouts [37].

There is both theoretical and empirical work suggesting that individuals work harder when in smaller groups [31,38–40]. For example, Cronin & Stumpe [40] showed that individuals in smaller groups have different strategies during collective actions, compared to individuals in larger groups. This work, with *Myrmecina nipponica* ant colonies shows that smaller groups work harder during assessment and transportation of nest-mates to new nest sites. *Temnothorax albipennis* colonies actively manage the size of the workforce during this initial search phase of an emigration, depending on their current housing conditions and when the collective scouting effort is lower, meaning the number of ants scouting is smaller, the individual effort in each bout increases.

Finding a suitable home is essential for an animal’s survival and reproductive success, hence distinguishing between a low-value and a high-value home is crucial. Several animals have been shown to be able to assess several nests and make the appropriate decision. For instance, female bats can rank nests according to their internal temperature [41], while for some birds food availability in the vicinity of a nesting cavity seems to be the deciding factor [42]. *Temnothorax albipennis* ants monitor attributes such as entrance size, height and light levels and for this study we used different combinations of these attributes to assemble a ranking of nest values [21].

One might imagine that the qualities of the different nests we have used are equally distant from one another; however that might not be the perception of the ants. It seems clear from these results that the difference between Poor and Satisfactory nests (different entrance sizes) and the difference between Good and Excellent nests (different levels of light exposure) are far greater than the differences between Satisfactory, Medium and Good. This may explain why in cases such as number of bouts, total path length and bout instantaneous speed, the fit to a cubic trend is also significant. Because, Satisfactory, Medium and Good nest values are closer to each other in comparison to either Poor or Excellent, there is a slight deviation in the overall linear trends. Previous research has shown which attributes colonies prefer in a nest and in what order, so we can definitely rank these five nests by value [21]. However, we do not know what absolute value ant colonies assign to each attribute and how much each nest differs, as perceived by the ants, from the others remains unclear.

This work builds on previous research showing how colonies can effectively respond to their environment and allocate the appropriate proportion of ants to a specific task, since all else being equal, the differences observed in the colonies have to be attributed to differences in nest value [24,25,43]. Here, we also show how individuals compensate for changes in the number of workers performing a task at a given time. That individuals of this species seem to work harder when in smaller groups is not new. Dornhaus *et al*. [31] showed that during nest emigration, workers of larger colonies had a lower workload in comparison to smaller colonies [31]. Furthermore, it has also been shown how variation in individual responses underpins the adaptive collective response of the colony albeit in discrete tasks such as fanning and carrying of nest-mates. Hence bumblebees will stop fanning once their nest temperature reaches the desired value and ants will cease carrying once they have arrived at their destination [30,44]. The novelty of this study is that we are focusing on scouting in the absence of a target nest and so the task is open-ended, and individuals must decide when to stop scouting before the task is completed. Furthermore, we show that the balance between workforce size and *per capita* work effort is actively controlled as a consequence of the environmental conditions. Increasing group size improves the ability to choose the most rewarding resources [45] and when the need to find a better resource is greater, colonies actively control the scouting effort by adjusting which individuals are actively scouting and how much work such individuals perform in each bout. Hence, the collective pattern observed might be the result of both variation in the amount of scouting and of behavioural flexibility of individual ants.

## Supplementary Material

doran_SF.docx contain supplementary figures

## Supplementary Material

doran_SI.docx contain all the code used for parameter calculation and statistical analysis

## Supplementary Material

bout_effort.txt

## Supplementary Material

collective_effort.txt contain the raw data

## Data accessibility

All data are available in the electronic supplementary material.
