# Supplementary material for: Exploration adjustment by ant colonies
Source: R Soc Open Sci. 2016 Jan 27;3(1):150533. doi: 10.1098/rsos.150533 (PMC4736935; doi:10.1098/rsos.150533)
Supplement: doran_SF.docx contain supplementary figures [file rsos150533supp1.docx]

Supplementary figures

***Ant colonies explore less but individuals search for longer when current housing conditions are better.***

**Effect of current nest value on the number of bouts:**


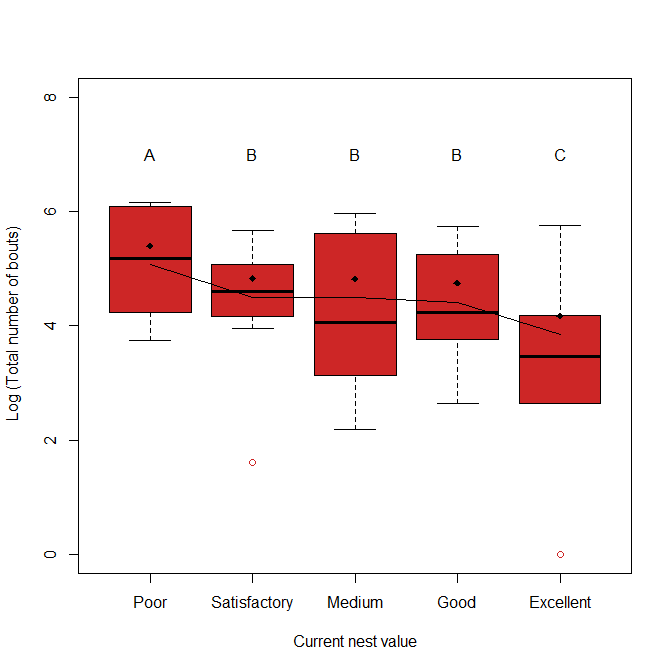


Figure S1 – Boxplot of the log of the number of bouts each colony performed when inhabiting the 5 different current nests. Black dots represent mean value and line represents the fitted values from the generalized linear mixed model. The fitted values show that even though the data fits significantly a cubic trend, the number of bouts does not increase and the overall trend is downwards. Here we show logged values because the generalized linear mixed model uses a log link. Calculation of fitted values can be found in the supplementary information (section f).

Goodness of fit of Generalized linear mixed model for number of bouts:


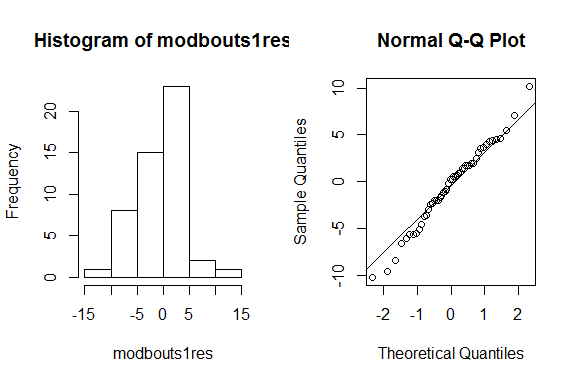


Figure S2 – Overall residuals for the model are approximately normal with a bit of deviation from normality on the tails (Q-Q plot). However Shapiro-Wilk normality test shows that this is very close to normality (W: 0.987, p = 0.8548, supplementary information section c).


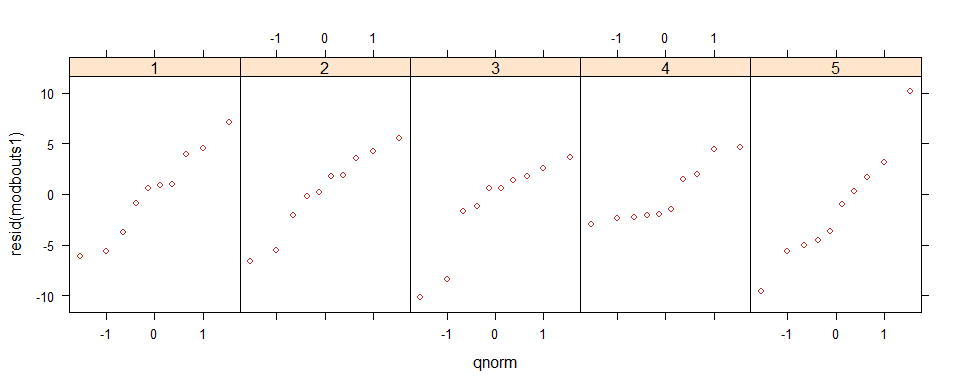


Figure S3 – A Q-Q plot for the residuals per nest quality (1 – 5 represent increasing nest quality)


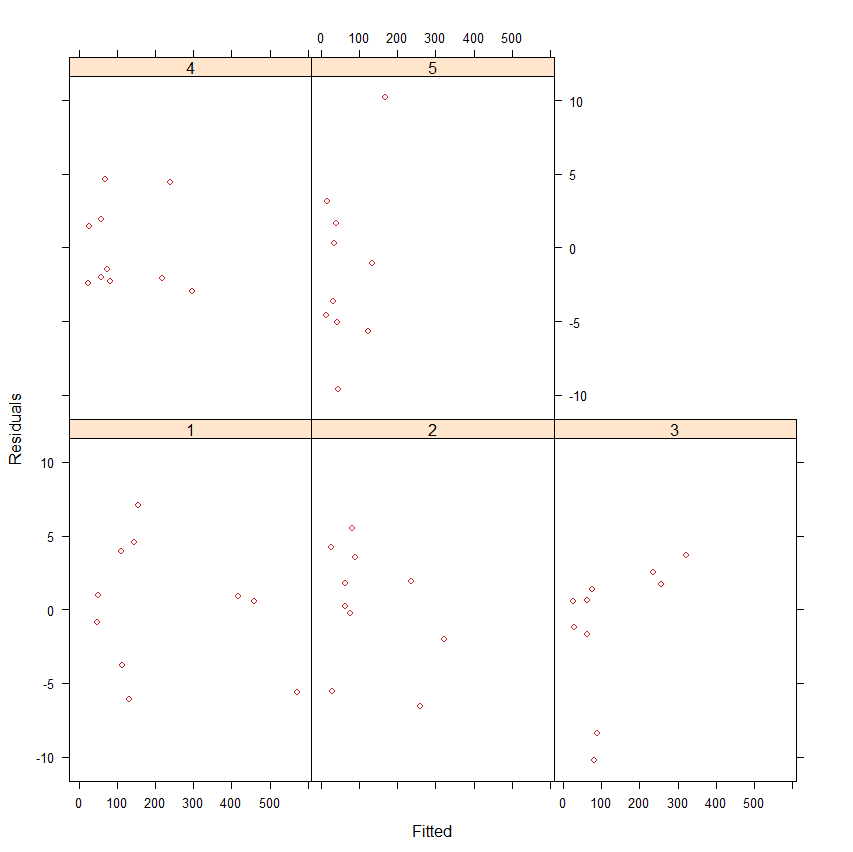


Figure S4 – Residuals-versus-fitted-values per nest value are approximately homogeneous.


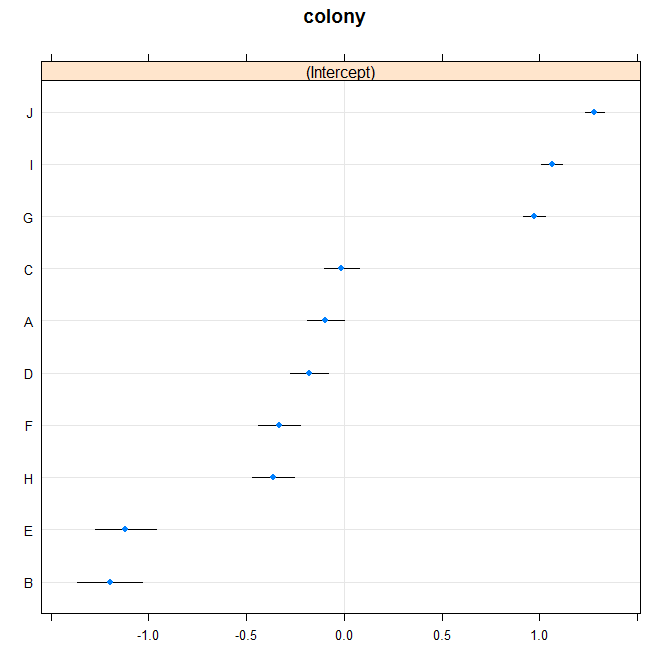


Figure S5 – Caterpillar plot for the effect of random factor in the model. The 95% interval of colony C touches zero but none of the 10 colonies straddle zero. This means that colonies deviate significantly from the overall intercept and hence inclusion of the random factor is necessary.

**Effect of current nest value on total amount of work:**


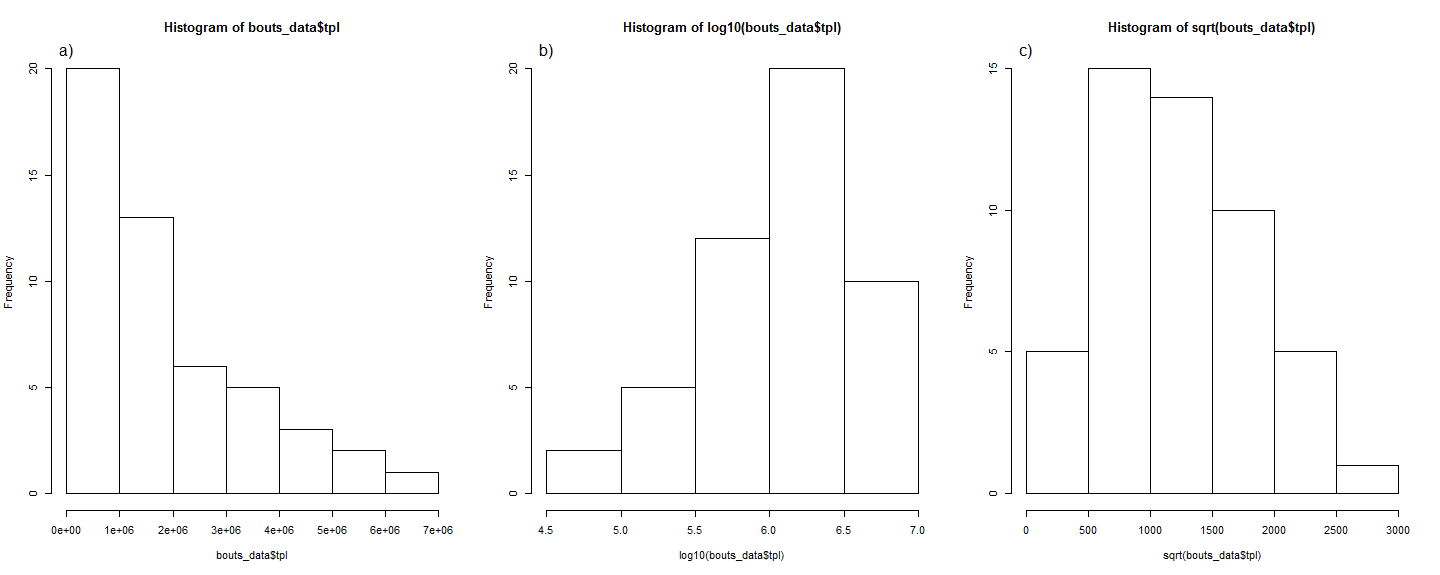


Figure S6 – Choosing the best transformation for the total path length to run the linear mixed model: a) Untransformed data; b) Logged data; c) Square root of the data. Both analysing visually and according to Shapiro-Wilk normality test (W = 0.9788, p = 0.8792; supplementary material section d) the transformation which is closer to normality is the square root.


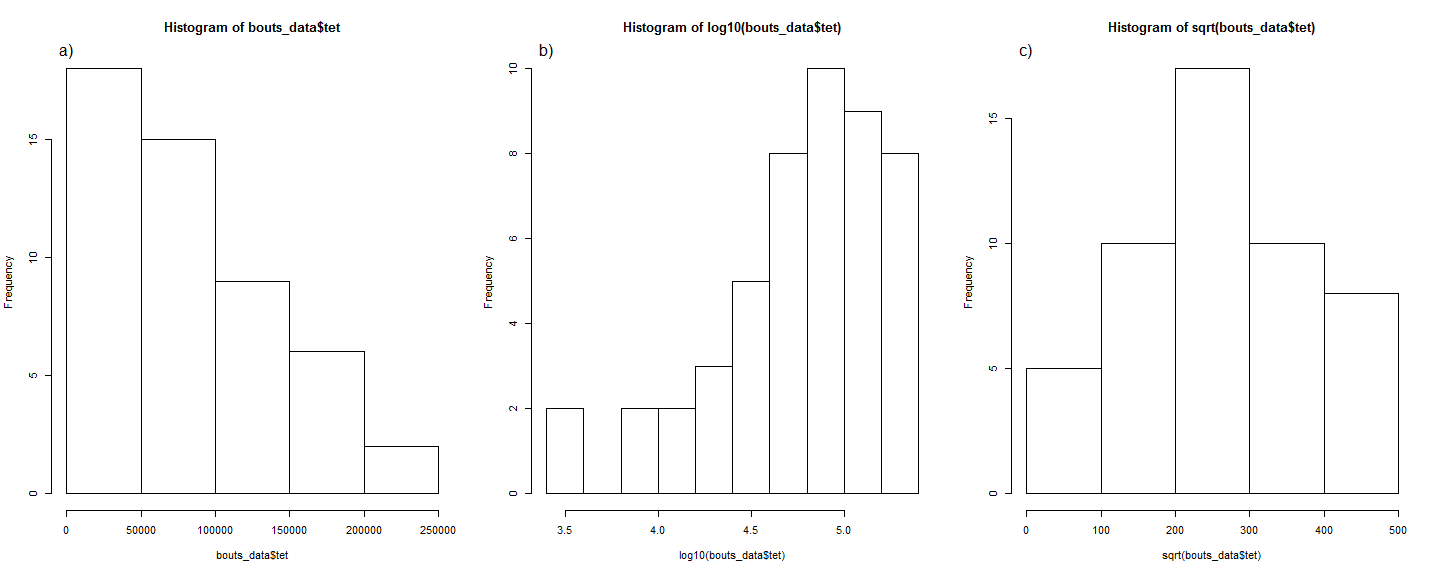


Figure S7 – Choosing the best transformation for the total exploration time to run the linear mixed model: a) Untransformed data; b) Logged data; c) Square root of the data. Both visually and according to Shapiro-Wilk normality test (W = 0.9879, p = 0.8865; supplementary material section e the transformation which is closer to normality is the square root.


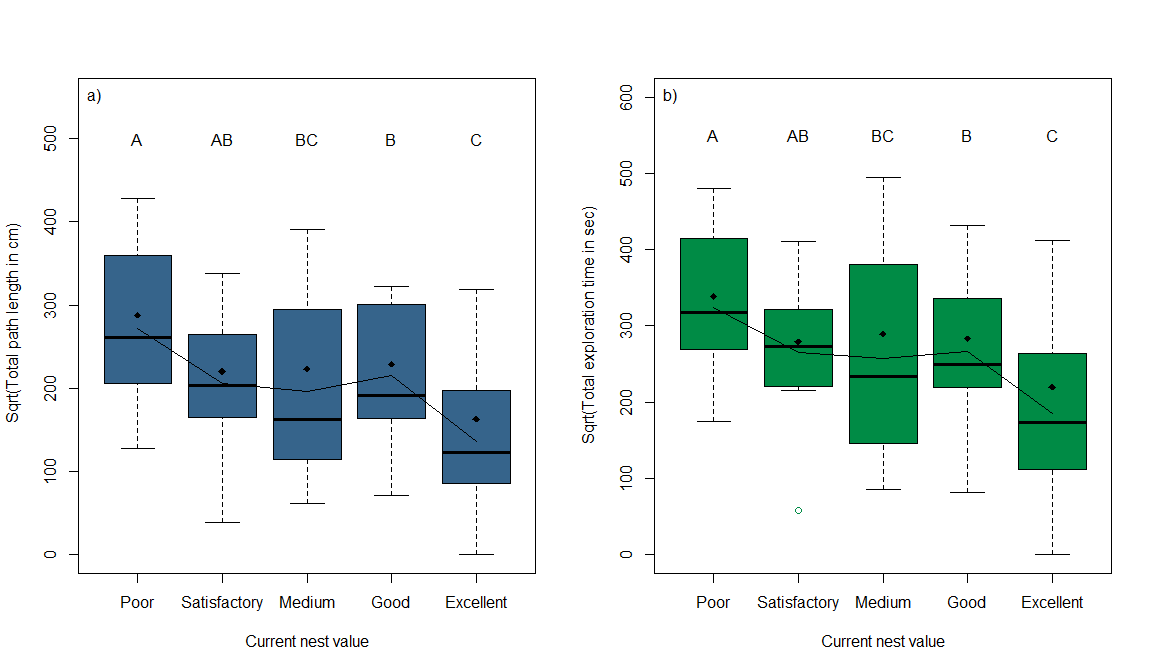


Figure S8 – Boxplot of the different measurements of collective effort for the different housing conditions: a) Square root of Total path length; b) Square root of Total exploration time. Black dots represent the mean and lines represent the fitted values from the mixed model. For path length we can see that there is an increase between Medium a Good current nests, which could explain why we get a significant p value for the fit of a cubic trend as well as a linear trend (p =1.35*10^-5^, 0.00679 for linear and cubic trend respectively). However the difference between the two current nests is not significant. Also the p value for the linear trend is substantially smaller.

Goodness of fit of linear mixed model for total path length:


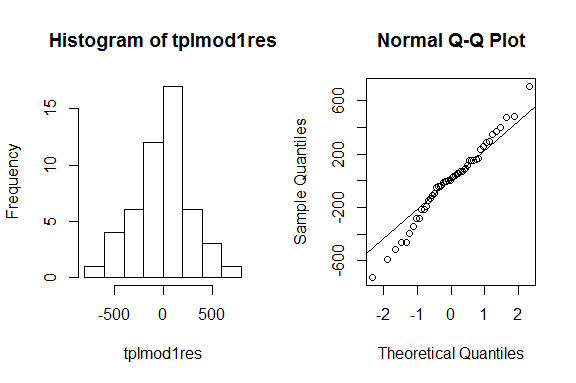


Figure S9 - Overall residuals for the model are approximately normal with a bit of deviation from normality on the tails (Q-Q plot). However Shapiro-Wilk normality test shows that this is very close to normality (W: 0.984, p = 0.7299, supplementary information section d).


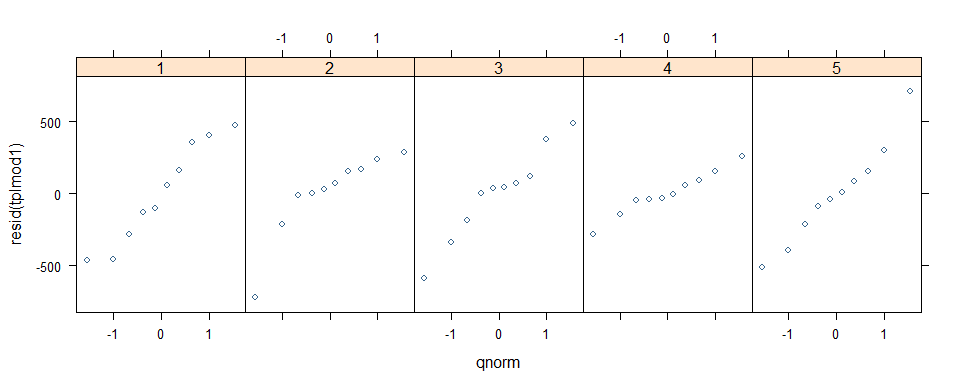


Figure S10 – Q-Q plot for the residuals per nest quality (1 to 5 represent increasing nest quality).


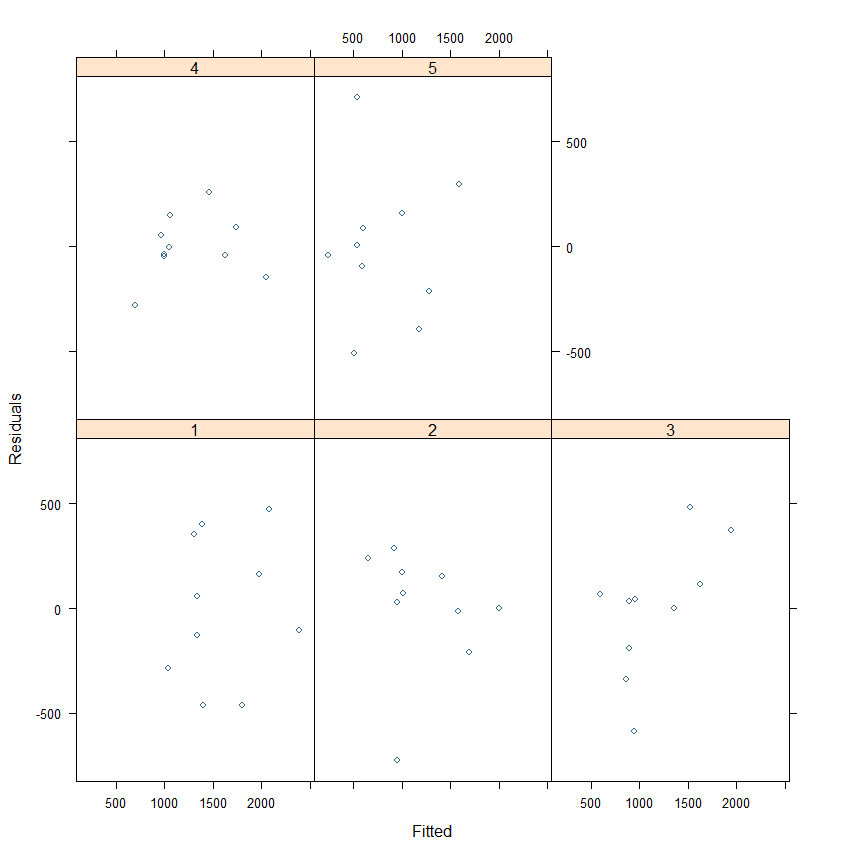


Figure S11 – Residuals versus fitted values for total path length reveal that residuals are approximately homogeneous.


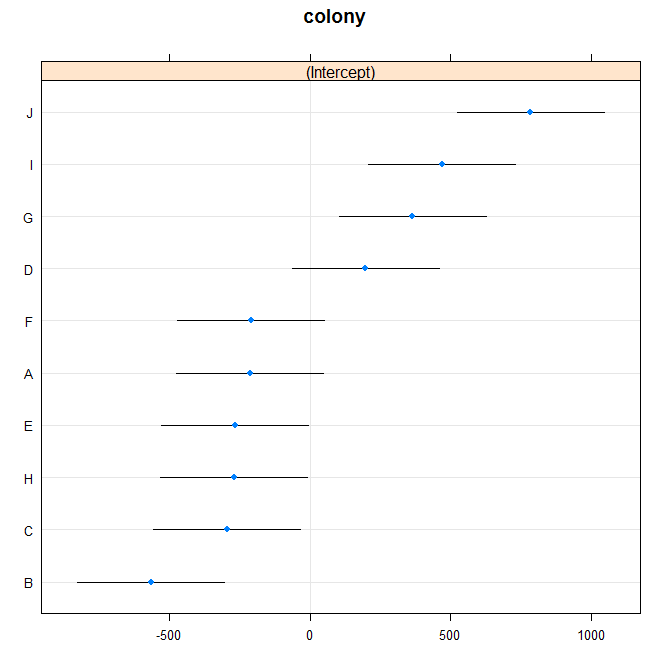


Figure S12 – Caterpillar plot for the effect of random factor in the model. The 95% interval of colonies A, D and F touch zero but none of the 10 colonies straddle zero. This means that colonies deviate significantly from the overall intercept and hence inclusion of the random factor is necessary.

Goodness of fit of linear mixed model for total exploration time:


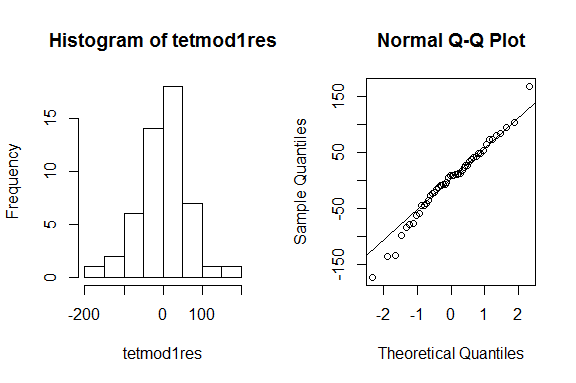


Figure S13 – Overall residuals for the model are approximately normal with a bit of deviation from normality on the tails (Q-Q plot). However Shapiro-Wilk normality test shows that this is very close to normality (W = 0.9819, p = 0.6329, supplementary information section e).


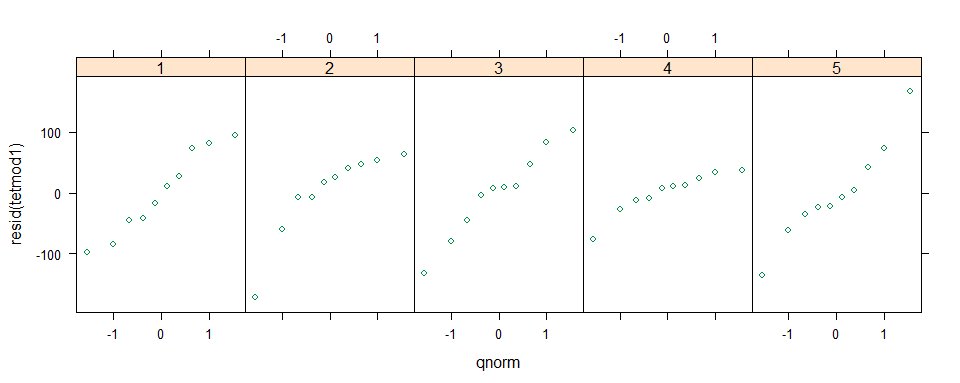


Figure S14 – Q-Q plot for the residuals per nest quality (1 to 5 represent increasing nest quality).


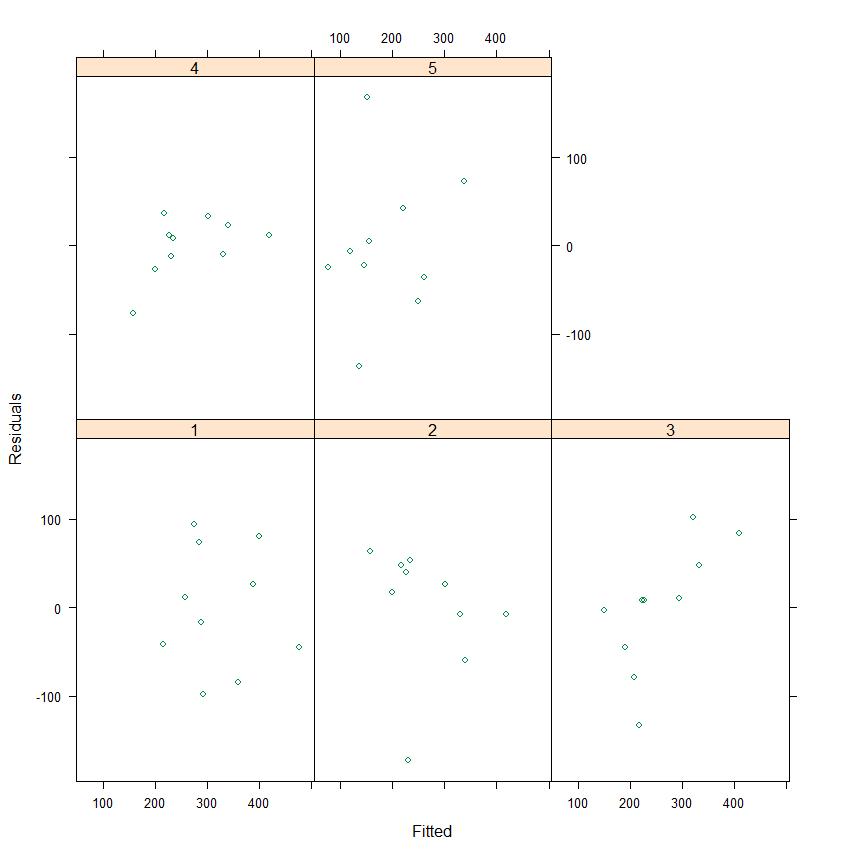


Figure S15 – Residuals versus fitted values reveal that residuals are approximately homogeneous.


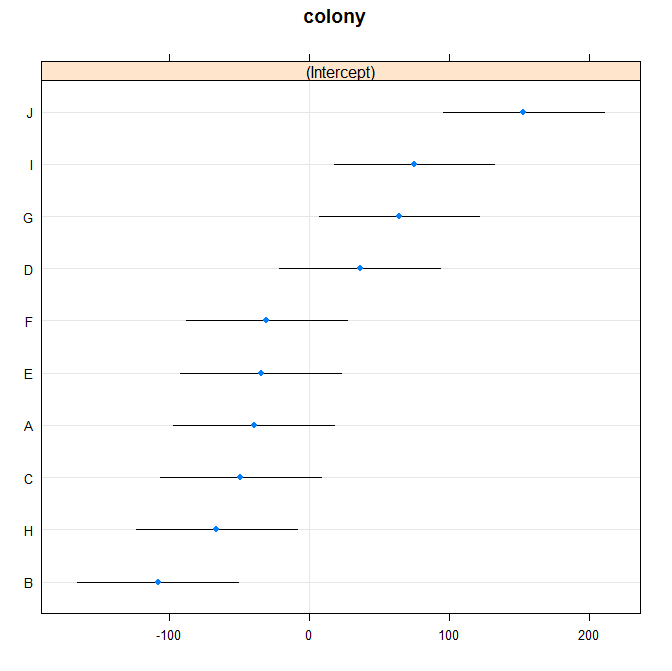


Figure S16 – Caterpillar plot for the effect of random factor in the model. The 95% interval of colonies A, C, D, E and F touch zero but none of the 10 colonies straddle zero. This means that colonies deviate significantly from the overall intercept and hence inclusion of the random factor is necessary.

**Distribution of work analysis:**

**
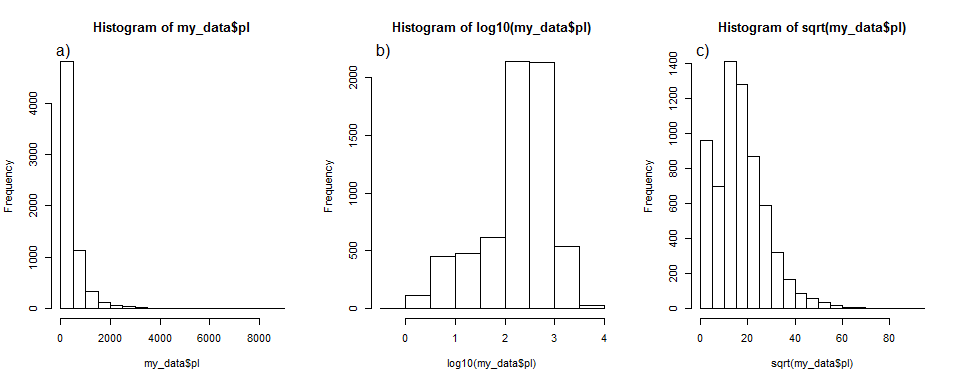
**

Figure S17 – Choosing the best transformation for the bout path length to run the linear mixed model: a) Untransformed data; b) Logged data; c) Square root of the data. We can’t do a normality test because the data set is too big. However the distribution of the log data does not look very far from normality.


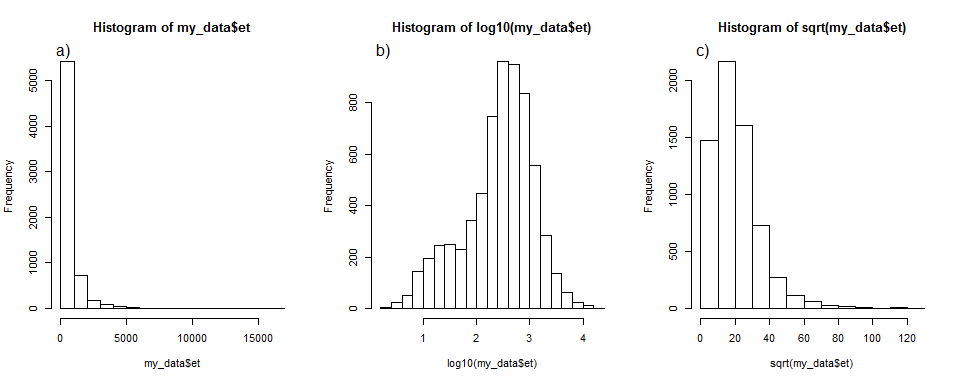


Figure S18 – Choosing the best transformation for the bout duration to run the linear mixed model: a) Untransformed data; b) Logged data; c) Square root of the data. We can’t do a normality test because the data set is too big. However the distribution of the log data does not look very far from normality.


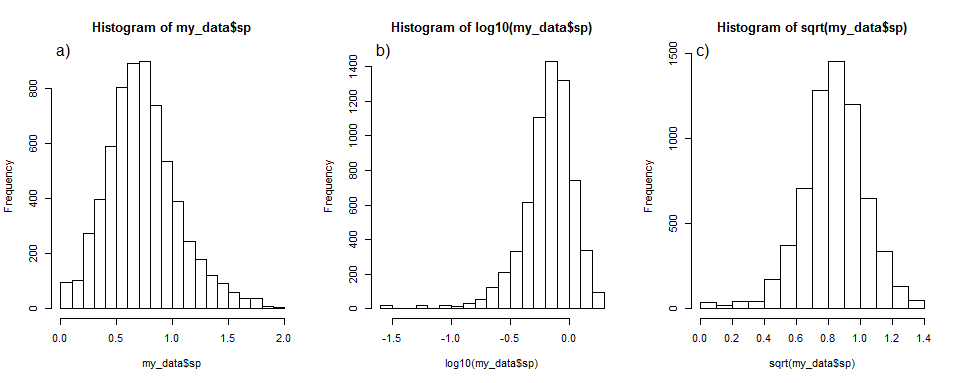


Figure S19 – Choosing the best transformation for the bout instantaneous speed to run the linear mixed model: a) Untransformed data; b) Logged data; c) Square root of the data. We can’t do a normality test because the data set is too big. However the distribution of the untransformed data does not look very far from normality.


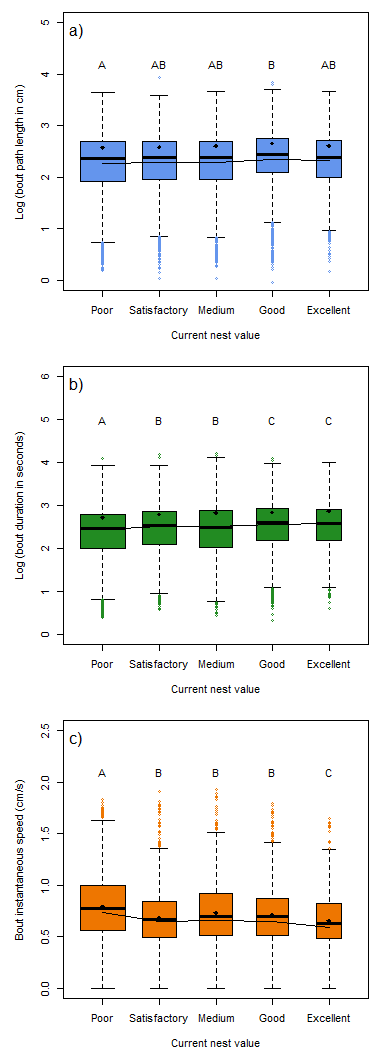


Figure S20 – Boxplot of the work performed in each exploration bout: a) Bout path length; b) Bout duration; c) Bout instant speed: Black dots represent the means and the line represents the fitted values from the mixed models.

Goodness of fit of linear mixed model for bout path length:


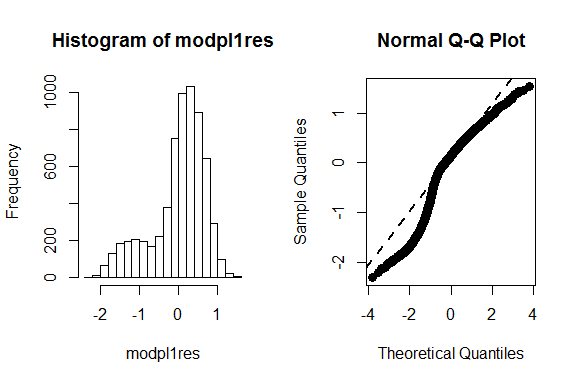


Figure S21 – Overall residuals for the model are approximately normal with a some deviation from normality on the tails (Q-Q plot). We are not able to use normality tests in this case due to very big sample size.


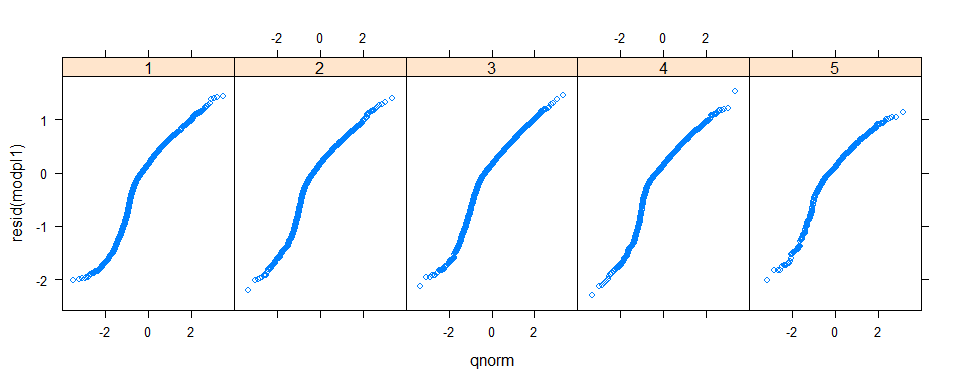


Figure S22 – Q-Q plot for the residuals per nest quality (1 to 5 represent increasing nest quality) reveals that most of the deviations from normality are in the extreme tails, particularly for the highest nest quality.


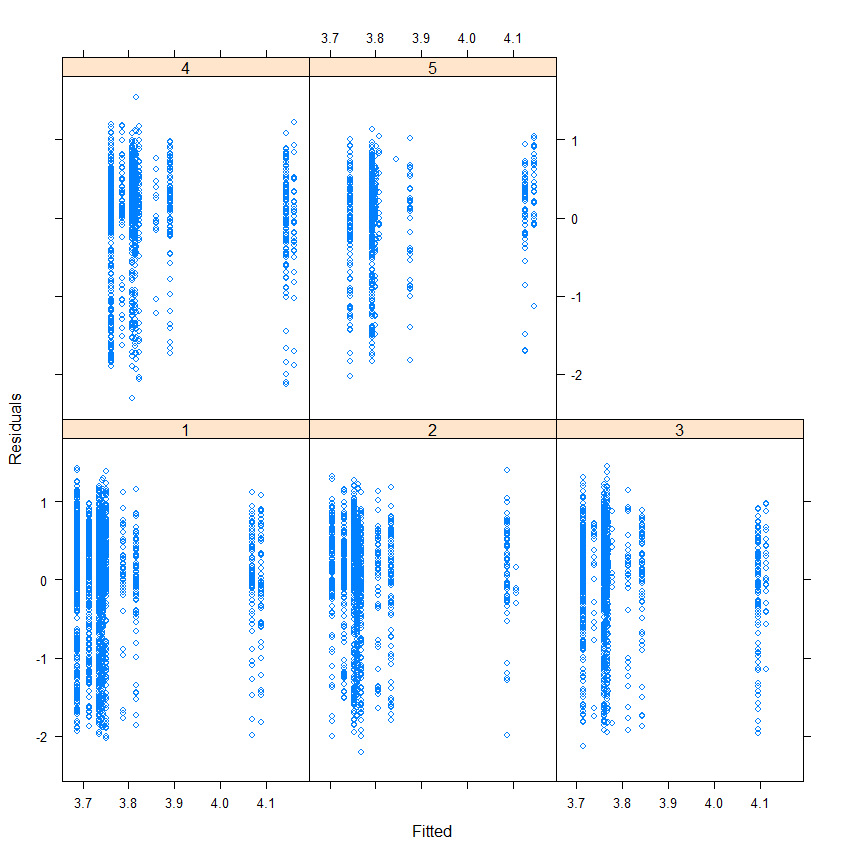


Figure S23 – Residuals versus fitted values reveal that residuals are approximately homogeneous.


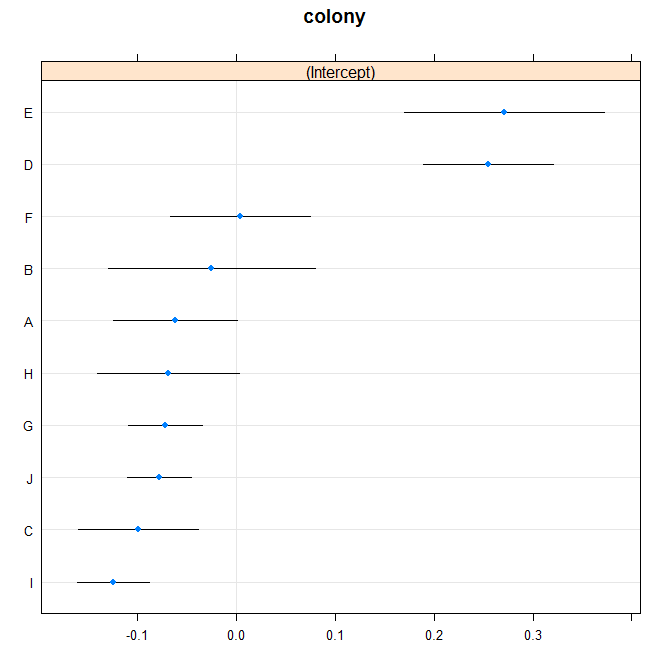


Figure S24 – Caterpillar plot for the effect of random factor in the model. The 95% interval of colonies B, F and H touch zero but none of the 10 colonies straddle zero. This means that colonies deviate significantly from the overall intercept and hence inclusion of the random factor is necessary.

Goodness of fit of linear mixed model for bout duration:


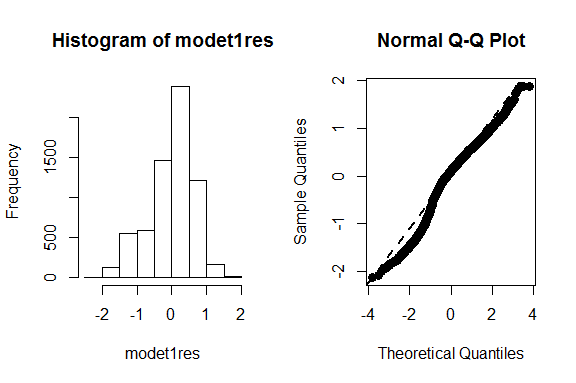


Figure S25 – Overall residuals for the model are approximately normal with some deviation from normality on the tails (Q-Q plot). We are not able to use normality tests in this case due to very big sample size.


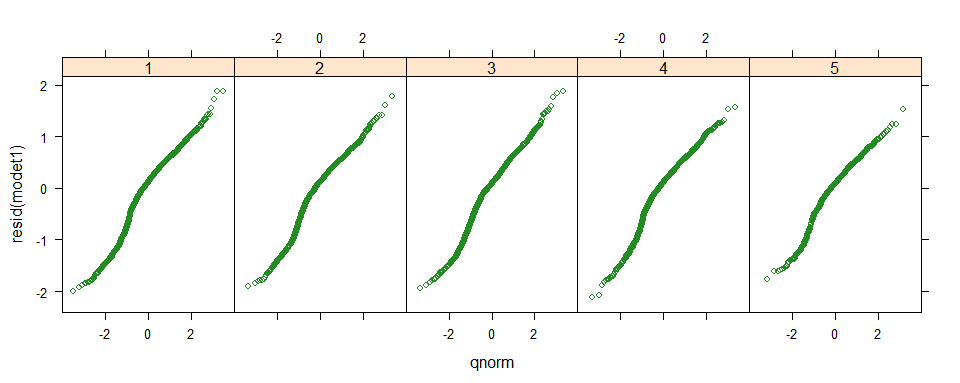


Figure S26 – Q-Q plot for the residuals per nest quality (1 to 5 represent increasing nest quality) reveals that most of the deviations from normality are in the extreme tails, particularly for the highest nest qualities, 4 and 5.


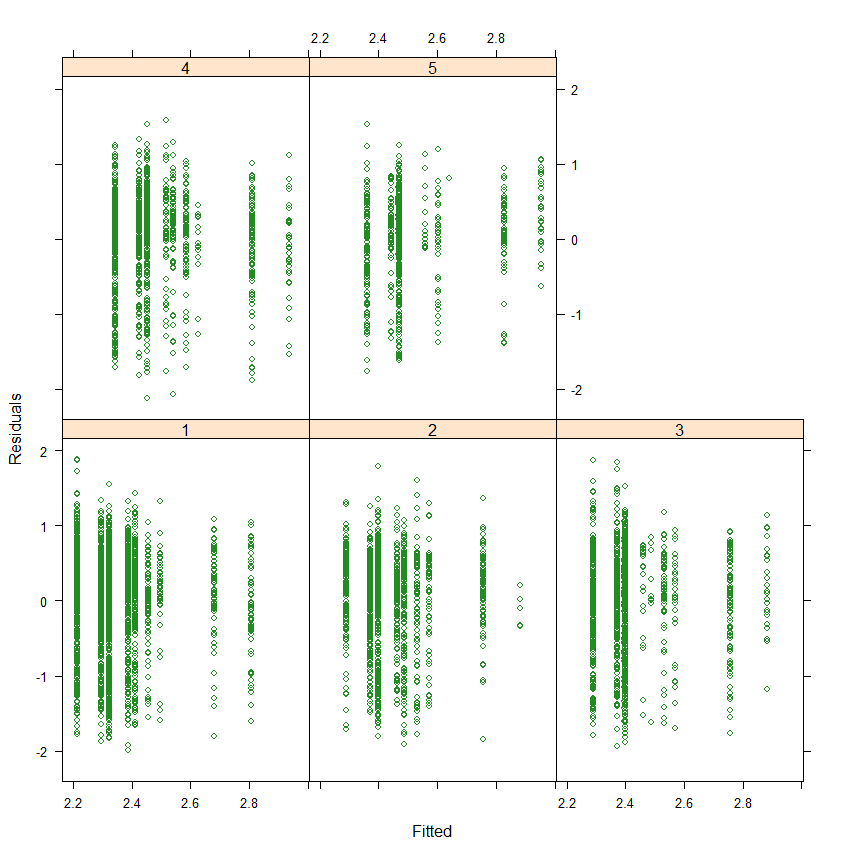


Figure S27 – Residuals versus fitted values reveal that residuals are approximately homogeneous.


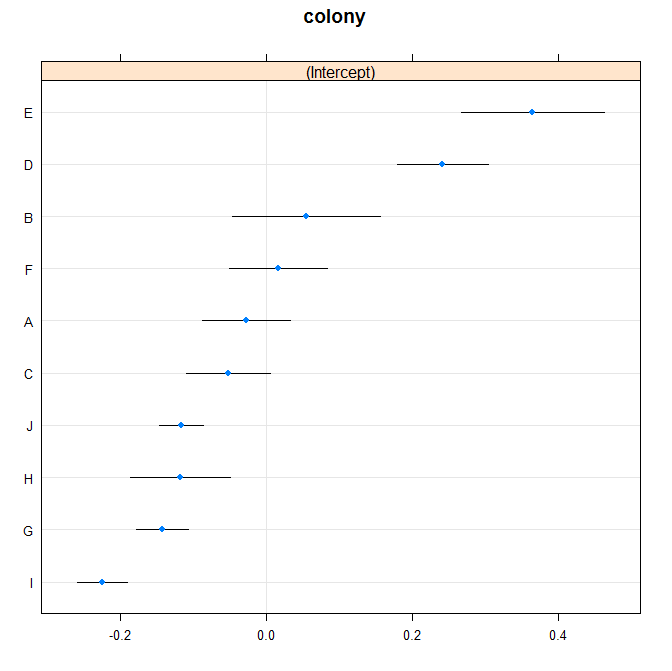


Figure S28 – Caterpillar plot for the effect of random factor in the model. The 95% interval of colonies A, B and F touch zero but none of the 10 colonies straddle zero. This means that colonies deviate significantly from the overall intercept and hence inclusion of the random factor is necessary.

Goodness of fit of linear mixed model for bout instantaneous speed:


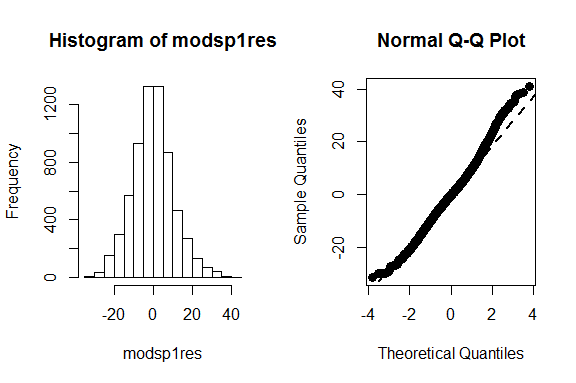


Figure S29 – Overall residuals for the model are approximately normal with a bit of deviation from normality on the tails (Q-Q plot). We are not able to use normality tests in this case due to very big sample size.


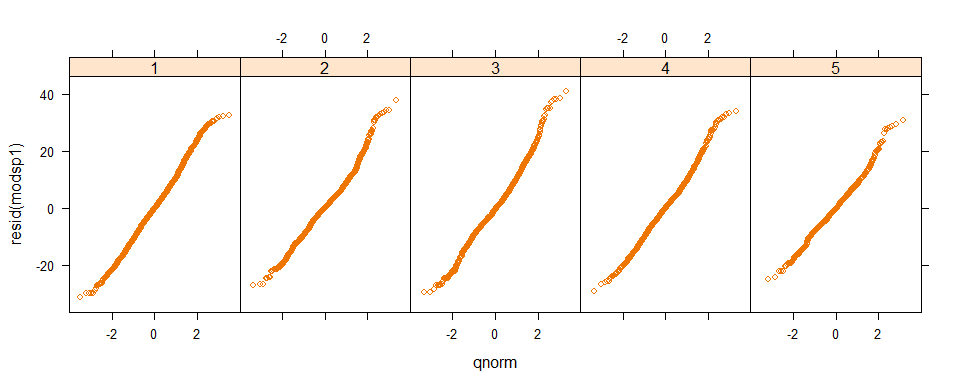


Figure S30 - Q-Q plot for the residuals per nest quality (1 to 5 represent increasing nest quality) reveals that most of the deviations from normality are in the extreme tails.


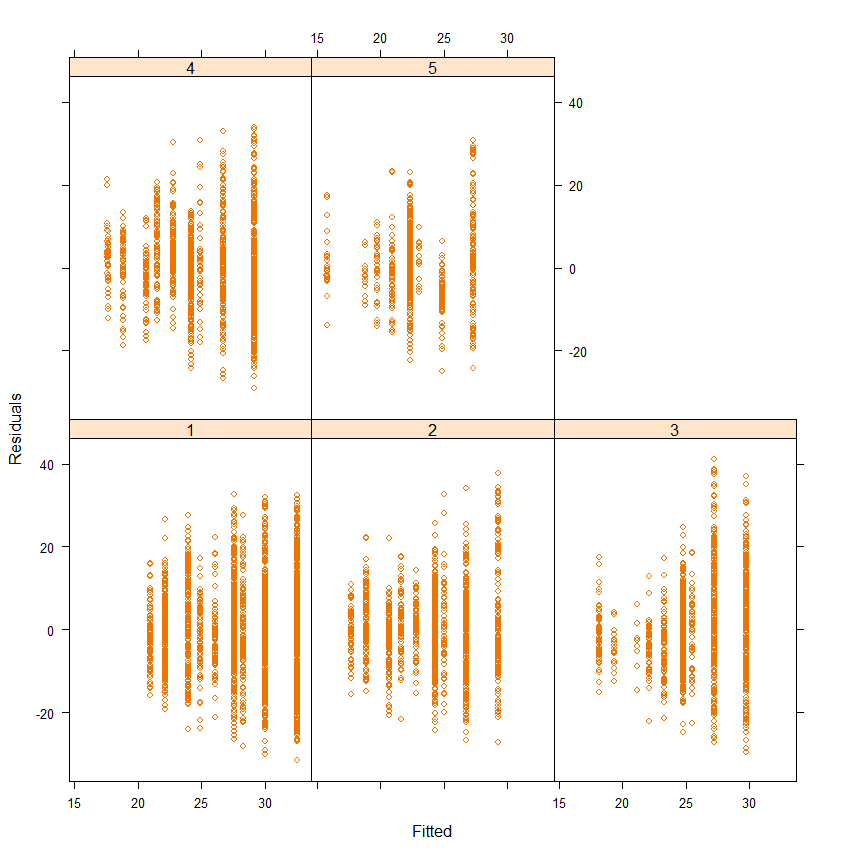


Figure S31 – Residuals versus fitted values reveal that residuals are approximately homogeneous


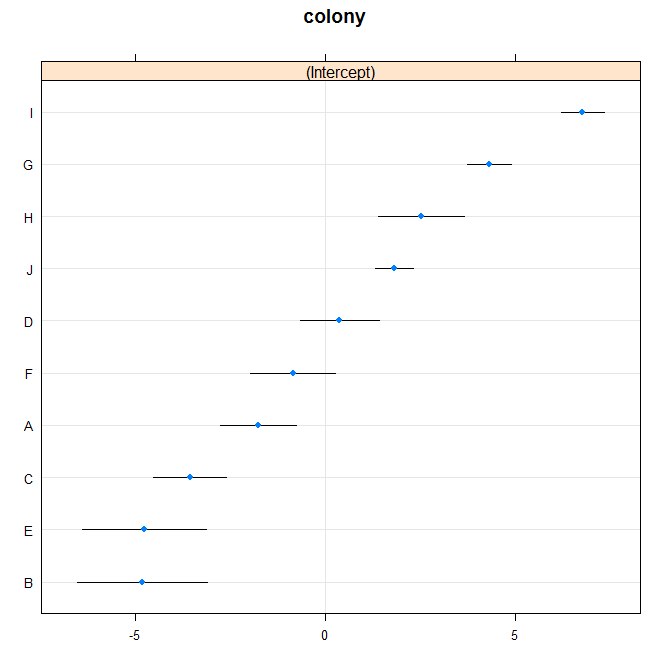


Figure S32 – Caterpillar plot for the effect of random factor in the model. The 95% interval of colonies D and F touch zero but none of the 10 colonies straddle zero. This means that colonies deviate significantly from the overall intercept and hence inclusion of the random factor is necessary.


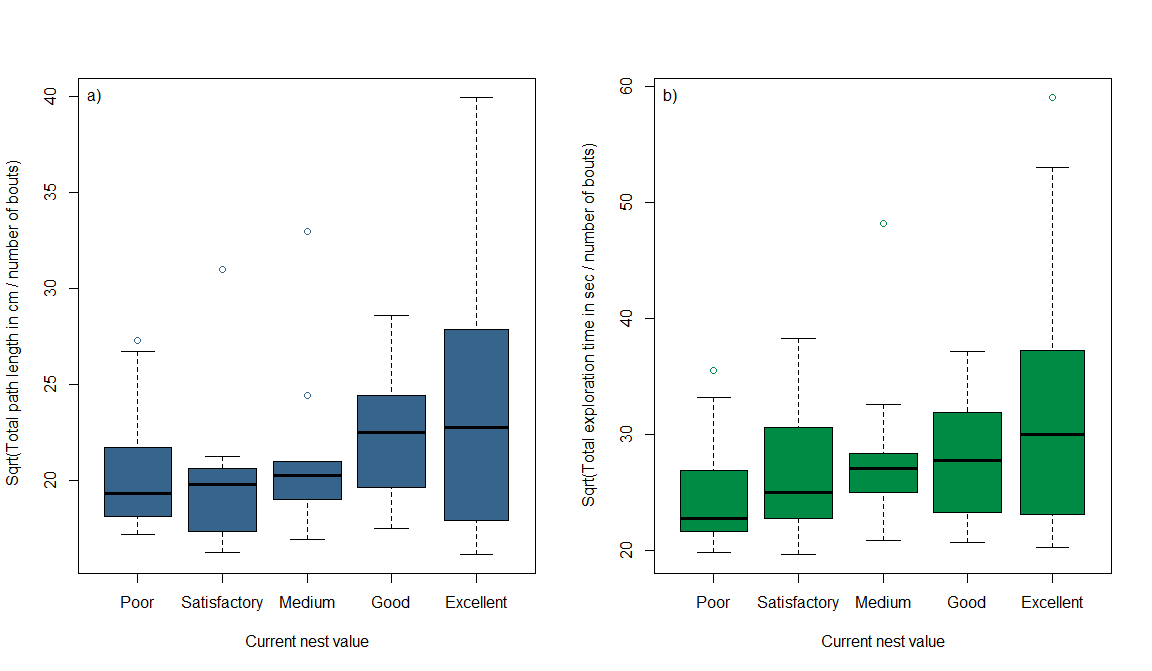


Figure S33 – Boxplot of the different measurements of collective effort for the different housing conditions divided by total number of bouts: a) Square root of Total path length; b) Square root of Total exploration time. A linear mixed model with each of the response variables divided by total number of bouts shows a significant fit to a positive linear trend (p = 0.0123 and 0.00157 for total path length and total exploration duration respectively)
