# Supplementary material for: Exploration adjustment by ant colonies
Source: R Soc Open Sci. 2016 Jan 27;3(1):150533. doi: 10.1098/rsos.150533 (PMC4736935; doi:10.1098/rsos.150533)
Supplement: doran_SI.docx contain all the code used for parameter calculation and statistical analysis [file rsos150533supp2.docx]

Supplementary information

***Ant colonies explore less but individuals search for longer when current housing conditions are better.***

1. Calculation of Bout path length, duration and instantaneous speed
2. Calculation of Number of bouts, Total path length and Total exploration time
3. Number of bouts analysis
4. Total path length analysis
5. Total exploration time analysis
6. Calculation of fitted values for Number of bouts, Total path length and Total exploration time
7. Bout path length analysis
8. Bout duration analysis
9. Bout instant speed analysis
10. Calculation of fitted values for Bout path length, duration and instantaneous speed
11. Total path length and total exploration time divided by number of bouts analysis

**Python code:**

1. Calculation of Bout path length, duration and instantaneous speed:

**import** glob

**import** numpy **as** np

**import** pylab **as** pl

**import** math

# from this we get a list of folders per day containing several trajectory text files

colony_day **=** glob**.**glob**(**'Col*/D*/*trajectories'**)**

##############################################################################################

##############################################################################################

##############################################################################################

day_to_value_dict **=** **{**'Colony A'**:** **[**0**,**2**,**1**,**4**,**5**,**3**],** 'Colony B'**:** **[**0**,**3**,**2**,**1**,**4**,**5**],** 'Colony C'**:** **[**0**,**1**,**4**,**2**,**3**,**5**],** 'Colony D'**:** **[**0**,**4**,**3**,**2**,**1**,**5**],** 'Colony E'**:** **[**0**,**5**,**3**,**1**,**4**,**2**],** 'Colony F'**:** **[**0**,**3**,**4**,**2**,**5**,**1**,**2**,**3**,**1**,**5**,**4**],** 'Colony G'**:** **[**0**,**4**,**5**,**3**,**1**,**2**,**3**,**4**,**2**,**1**,**5**],** 'Colony H'**:** **[**0**,**5**,**2**,**1**,**3**,**4**,**1**,**5**,**4**,**3**,**2**],** 'Colony I'**:** **[**0**,**1**,**3**,**4**,**2**,**5**,**4**,**1**,**5**,**2**,**3**],** 'Colony J'**:** **[**0**,**2**,**1**,**5**,**4**,**3**,**5**,**2**,**4**,**1**,**3**]}**

nest_value **=** **{**1**:**'Poor'**,** 2**:** 'Satisfactory'**,** 3**:** 'Medium'**,** 4**:** 'Good'**,** 5**:**'Deluxe'**}**

pixel_mm **=** **{**'A'**:** **(**1274**,** 1251**,** 1262**,** 1284**,** 1288**),** 'B'**:** **(**1235**,** 1252**,** 1299**,** 1291**,** 1285**),** 'C'**:** **(**1246**,** 1255**,** 1277**,** 1278**,** 0**),** 'D'**:** **(**1210**,** 1310**,** 1300**,** 1303**,** 1288**),** 'E'**:** **(**1247**,** 1275**,** 1287**,** 1293**,** 1282**),** 'F'**:** **(**1300**,** 1308**,** 1302**,** 1301**,** 1298**),** 'G'**:** **(**1290**,** 1301**,** 1306**,** 1302**,** 1298**),** 'H'**:** **(**1287**,** 1300**,** 1301**,** 1298**,** 1297**),** 'I'**:** **(**1311**,** 1304**,** 1300**,** 1299**,** 1302**),** 'J'**:** **(**1300**,** 1306**,** 1300**,** 1297**,** 1300**)}**

##############################################################################################

##############################################################################################

##############################################################################################

output_file **=** open**(**bout_effort.txt'**,**'w'**)**

output_file**.**write**(**'Colony\tDay\tTrajectory\tValue\tPLength\tExplTime\tSpeed\n'**)**

**for** traj_folder **in** colony_day**:**

**print** '*****************'

**print** traj_folder

**print** '*****************'

trajectories **=** glob**.**glob**(**traj_folder **+** '/*.txt'**)**

**print** trajectories

###### calculating entrance

**for** traj **in** trajectories**:**

**print** 'Working on:'

**print** traj

data **=** open**(**traj**,** 'r'**).**readlines**()[**1**:]**

plength_list **=** **[]**

speed_list **=** **[]**

n **=** 0

speed_temp_list **=** list**()**

**for** i**,** line **in** enumerate**(**data**):**

parts **=** line**.**strip**().**split**()**

parts **=** **[**float**(**p**)** **for** p **in** parts**]**

**if** i **==** 0**:**

x_prev **=** parts**[**1**]**

y_prev **=** parts**[**2**]**

**if** i **>** 0**:**

x **=** parts**[**1**]**

y **=** parts**[**2**]**

plength **=** math**.**sqrt**(** **(**x **-** x_prev**)****2 **+** **(**y **-** y_prev**)****2 **)**

plength_list**.**append**(**plength**)**

x_prev **=** x

y_prev **=** y

**if** n **<** 50**:**

speed_temp_list**.**append**(**plength**)**

n **+=**1

**else:**

speed_list**.**append**(**sum**(**speed_temp_list**))**

speed_temp_list **=** list**()**

n **=** 0

colony **=** traj**[**7**]**

day **=** traj**[**13**:**15**].**strip**()**

trajectory **=** traj**[**51**:-**4**]**

value **=** str**(**day_to_value_dict**[**traj_folder**[**0**:**8**]][**int**(**traj_folder**[**13**])])**

bout_plength **=** **(**sum**(**plength_list**)***37**)/**pixel_mm**[**colony**][**int**(**day**)-**1**]**

bout_duration **=** len**(**plength_list**)/**50.0

speed **=** **(**np**.**median**(**speed_list**)***37**)/**pixel_mm**[**colony**][**int**(**day**)-**1**]**

output_file**.**write**(**colony **+** '\t' **+** day **+** '\t' **+** trajectory **+** '\t' **+** value **+** '\t' **+** str**(**bout_plength**)** **+** '\t' **+** str**(**bout_duration**)** **+** '\t' **+** str**(**speed**)** **+** '\n'**)**

output_file**.**close**()**

**print** 'Done'

1. Calculation of Number of bouts, Total path length and Total exploration time:

**import** numpy **as** np

**import** numpy **as** np

**import** pylab **as** pl

**class** **Result(**object**):**

**def** __init__ **(**self**,** line**):**

**(**self**.**colony**,** self**.**day**,** self**.**traj**,** self**.**value**,** self**.**plength**,** self**.**time**,** self**.**speed**)** **=** line**.**split**()**

self**.**day **=** int**(**self**.**day**)**

self**.**value **=** int**(**self**.**value**)**

self**.**plength **=** float**(**self**.**plength**)**

self**.**time **=** float**(**self**.**time**)**

self**.**speed **=** float**(**self**.**speed**)**

**class** **Results** **(**object**):**

**def** __init__**(**self**,** filename**):**

self**.**results **=** list**()**

lines **=** open**(**filename**,** 'r'**).**readlines**()**

lines **=** lines**[**1**:]**

**for** line **in** lines**:**

**if** line**.**strip**():**

self**.**results**.**append**(** Result**(**line**))**

**def** get **(**self**,** colony **=** **None,** day **=** **None,** value **=** **None):**

results **=** self**.**results**[:]**

**if** colony **is** **not** **None:**

results **=** **[**r **for** r **in** results **if** r**.**colony **==** colony**]**

**if** day **is** **not** **None:**

results **=** **[**r **for** r **in** results **if** r**.**day **==** day**]**

**if** value **is** **not** **None:**

results **=** **[**r **for** r **in** results **if** r**.**value **==** value**]**

colonies **=** np**.**array**([**r**.**colony **for** r **in** results**])**

days **=** np**.**array**([**r**.**day **for** r **in** results**])**

trajs **=** np**.**array**([**r**.**traj **for** r **in** results**])**

values **=** np**.**array**([**r**.**value **for** r **in** results**])**

plengths **=** np**.**array**([**r**.**plength **for** r **in** results**])**

times **=** np**.**array**([**r**.**time **for** r **in** results**])**

speeds **=** np**.**array**([**r**.**speed **for** r **in** results**])**

**return** **(**colonies**,** days**,** trajs**,** values**,** plengths**,** times**,** speeds**)**

###########################################################

###########################################################

###########################################################

output_file **=** open**(**'collective_effort.txt'**,** 'w'**)**

output_file**.**write**(**'Colony\tDay\tValue\tBouts\tTotal_pathlength\tTotal_time\n'**)**

colonies **=** **[**'A'**,**'B'**,**'C'**,**'D'**,**'E'**,**'F'**,**'G'**,**'H'**,**'I'**,**'J'**]**

values **=** **[**1**,**2**,**3**,**4**,**5**]**

all_results **=** Results**(**'bout_effort.txt'**)**

**for** col **in** colonies**:**

**for** val **in** values**:**

**if** col **==** 'C' **and** val **==** 5**:**

**pass**

**else:**

data **=** all_results**.**get**(**colony **=** col**,** value **=** val**)**

day **=** str**(**data**[**1**][**0**])**

bouts **=** str**(**len**(**data**[**0**]))**

pathlength_total **=** str**(**sum**(**data**[**4**]))**

time_total **=** str**(**sum**(**data**[**5**]))**

output_file**.**write**(**col **+** '\t' **+** day **+** '\t' **+** str**(**val**)** **+** '\t' **+** bouts **+** '\t' **+** pathlength_total **+** '\t' **+** time_total **+** '\n'**)**

output_file**.**close**()**

**print** 'Done'

###########################################################

**R code:**

Loading libraries and importing data:

library**(**lattice**)**

library**(**gplots**)**

library**(**lmerTest**)**

bouts_data **<-** read.table**(**file **=** "collective_effort.txt", header **=** T**)**

colnames**(**bouts_data**)** **<-** c**(**"colony", "day", "treat", "bouts", "tpl", "tet"**)**

bouts_data**$**colony **<-** factor**(**bouts_data**$**colony, levels**=**c**(**"A","B","C","D","E","F","G","H","I","J"**)**, ordered **=** F**)**

bouts_data**$**treat **<-** factor**(**bouts_data**$**treat, levels**=**c**(**"1","2","3","4","5"**)**, ordered **=** T**)** # current nest value

bouts_data**$**bouts **<-** as.numeric**(**bouts_data**$**bouts**)** # Number of bouts

bouts_data**$**tpl **<-** as.numeric**(**bouts_data**$**tpl**)** # Total path length

bouts_data**$**tet **<-** as.numeric**(**bouts_data**$**tet**)** # Total exploration time

####

my_data **<-** read.table**(**file **=** "bout_effort.txt", header **=** T**)**

colnames**(**my_data**)** **<-** c**(**"colony", "day", "traj", "treat", "pl", "et", "dfe", "sp", "zerosp", "Meansl"**)**

my_data**$**colony **<-** factor**(**my_data**$**colony, levels**=**c**(**"A","B","C","D","E","F","G","H","I","J"**)**, ordered **=** F**)**

my_data**$**traj **<-** factor**(**my_data**$**traj**)** # Trajectory ID

my_data**$**treat **<-** factor**(**my_data**$**treat, levels**=**c**(**"1","2","3","4","5"**)**, ordered **=** T**)** # Current nest value

my_data**$**pl **<-** as.numeric**(**my_data**$**pl**)** # Bout path length

my_data**$**et **<-** as.numeric**(**my_data**$**et**)** # Bout duration

my_data**$**sp **<-** as.numeric**(**my_data**$**sp**)** # Bout instantaneous speed

1. Number of bouts analysis:

#### Models

modbouts0 **<-** glmer**(**bouts **~** **(**1**|**colony**)**, data **=** bouts_data, family **=** 'poisson'**)**

modbouts1 **<-** glmer**(**bouts **~** treat **+** **(**1**|**colony**)**, data **=** bouts_data, family **=** 'poisson'**)**

summary**(**modbouts1**)**

**--------------------------------------------------------------------------------**

Generalized linear mixed model fit by maximum likelihood **(**Laplace

Approximation**)** **[**glmerMod**]**

Family**:** poisson **(** log **)**

Formula**:** bouts **~** treat **+** **(**1 **|** colony**)**

Data**:** bouts_data

AIC BIC logLik deviance df.resid

1282.5 1294.0 **-**635.3 1270.5 44

Scaled residuals**:**

Min 1Q Median 3Q Max

**-**7.9975 **-**2.7177 0.2405 2.0151 11.4491

Random effects**:**

Groups Name Variance Std.Dev.

colony **(**Intercept**)** 0.6727 0.8202

Number of obs**:** 50, groups**:** colony, 10

Fixed effects**:**

Estimate Std. Error z value Pr**(>|**z**|)**

**(**Intercept**)** 4.46207 0.25996 17.164 **<**2e**-**16 *******

treat.L **-**0.80009 0.03106 **-**25.756 **<**2e**-**16 *******

treat.Q **-**0.02059 0.03033 **-**0.679 0.497

treat.C **-**0.33608 0.02945 **-**11.413 **<**2e**-**16 *******

treat**^**4 0.02319 0.02872 0.807 0.419

**---**

Signif. codes**:** 0 ‘*******’ 0.001 ‘******’ 0.01 ‘*****’ 0.05 ‘.’ 0.1 ‘ ’ 1

Correlation of Fixed Effects**:**

**(**Intr**)** tret.L tret.Q tret.C

treat.L 0.018

treat.Q 0.005 0.385

treat.C 0.008 0.071 0.219

treat**^**4 0.001 0.081 0.036 0.073

anova**(**modbouts0, modbouts1**)**

**--------------------------------------------------------------------------------**

Data**:** bouts_data

Models**:**

modbouts0**:** bouts **~** **(**1 **|** colony**)**

modbouts1**:** bouts **~** treat **+** **(**1 **|** colony**)**

Df AIC BIC logLik deviance Chisq Chi Df Pr**(>**Chisq**)**

modbouts0 2 2219.1 2222.9 **-**1107.53 2215.1

modbouts1 6 1282.5 1294.0 **-**635.26 1270.5 944.54 4 **<** 2.2e**-**16 *******

**---**

Signif. codes**:** 0 ‘*******’ 0.001 ‘******’ 0.01 ‘*****’ 0.05 ‘.’ 0.1 ‘ ’ 1

##Residuals

shapiro.test**(**resid**(**modbouts1**))**

**--------------------------------------------------------------------------------**

Shapiro**-**Wilk normality test

data**:** resid**(**modbouts1**)**

W **=** 0.987, p**-**value **=** 0.8548

#Contrats

**(**contrasts**(**bouts_data**$**treat**)** **<-** contr.treatment**(**levels**(**bouts_data**$**treat**)**,base**=**5**))**

modbouts1.5 **<-** glmer**(**bouts **~** treat **+** **(**1**|**colony**)**, data **=** bouts_data, family **=** 'poisson'**)**

summary**(**modbouts1.5**)**

**-----------------------------------------------------------------------------------------**

Generalized linear mixed model fit by maximum likelihood **(**Laplace

Approximation**)** **[**glmerMod**]**

Family**:** poisson **(** log **)**

Formula**:** bouts **~** treat **+** **(**1 **|** colony**)**

Data**:** bouts_data

AIC BIC logLik deviance df.resid

1282.5 1294.0 **-**635.3 1270.5 44

Scaled residuals**:**

Min 1Q Median 3Q Max

**-**7.9975 **-**2.7177 0.2404 2.0151 11.4490

Random effects**:**

Groups Name Variance Std.Dev.

colony **(**Intercept**)** 0.6727 0.8202

Number of obs**:** 50, groups**:** colony, 10

Fixed effects**:**

Estimate Std. Error z value Pr**(>|**z**|)**

**(**Intercept**)** 3.84153 0.26258 14.63 **<**2e**-**16 *******

treat1 1.22459 0.04467 27.42 **<**2e**-**16 *******

treat2 0.65541 0.04840 13.54 **<**2e**-**16 *******

treat3 0.64817 0.04846 13.38 **<**2e**-**16 *******

treat4 0.57449 0.04909 11.70 **<**2e**-**16 *******

**---**

Signif. codes**:** 0 ‘*******’ 0.001 ‘******’ 0.01 ‘*****’ 0.05 ‘.’ 0.1 ‘ ’ 1

Correlation of Fixed Effects**:**

**(**Intr**)** treat1 treat2 treat3

treat1 **-**0.131

treat2 **-**0.121 0.713

treat3 **-**0.121 0.712 0.657

treat4 **-**0.120 0.703 0.649 0.648

**(**contrasts**(**bouts_data**$**treat**)** **<-** contr.treatment**(**levels**(**bouts_data**$**treat**)**,base**=**4**))**

modbouts1.4 **<-** glmer**(**bouts **~** treat **+** **(**1**|**colony**)**, data **=** bouts_data, family **=** 'poisson'**)**

summary**(**modbouts1.4**)**

**-----------------------------------------------------------------------------------------**

Generalized linear mixed model fit by maximum likelihood **(**Laplace

Approximation**)** **[**glmerMod**]**

Family**:** poisson **(** log **)**

Formula**:** bouts **~** treat **+** **(**1 **|** colony**)**

Data**:** bouts_data

AIC BIC logLik deviance df.resid

1282.5 1294.0 **-**635.3 1270.5 44

Scaled residuals**:**

Min 1Q Median 3Q Max

**-**7.9975 **-**2.7177 0.2405 2.0151 11.4491

Random effects**:**

Groups Name Variance Std.Dev.

colony **(**Intercept**)** 0.6727 0.8202

Number of obs**:** 50, groups**:** colony, 10

Fixed effects**:**

Estimate Std. Error z value Pr**(>|**z**|)**

**(**Intercept**)** 4.41604 0.26129 16.901 **<**2e**-**16 *******

treat1 0.65009 0.03635 17.884 **<**2e**-**16 *******

treat2 0.08091 0.04085 1.981 0.0476 *****

treat3 0.07367 0.04092 1.800 0.0718 .

treat5 **-**0.57450 0.04909 **-**11.702 **<**2e**-**16 *******

**---**

Signif. codes**:** 0 ‘*******’ 0.001 ‘******’ 0.01 ‘*****’ 0.05 ‘.’ 0.1 ‘ ’ 1

Correlation of Fixed Effects**:**

**(**Intr**)** treat1 treat2 treat3

treat1 **-**0.091

treat2 **-**0.081 0.585

treat3 **-**0.081 0.584 0.519

treat5 **-**0.068 0.486 0.433 0.432

**(**contrasts**(**bouts_data**$**treat**)** **<-** contr.treatment**(**levels**(**bouts_data**$**treat**)**,base**=**3**))**

modbouts1.3 **<-** glmer**(**bouts **~** treat **+** **(**1**|**colony**)**, data **=** bouts_data, family **=** 'poisson'**)**

summary**(**modbouts1.3**)**

**-----------------------------------------------------------------------------------------**

Generalized linear mixed model fit by maximum likelihood **(**Laplace

Approximation**)** **[**glmerMod**]**

Family**:** poisson **(** log **)**

Formula**:** bouts **~** treat **+** **(**1 **|** colony**)**

Data**:** bouts_data

AIC BIC logLik deviance df.resid

1282.5 1294.0 **-**635.3 1270.5 44

Scaled residuals**:**

Min 1Q Median 3Q Max

**-**7.9975 **-**2.7177 0.2405 2.0151 11.4491

Random effects**:**

Groups Name Variance Std.Dev.

colony **(**Intercept**)** 0.6727 0.8202

Number of obs**:** 50, groups**:** colony, 10

Fixed effects**:**

Estimate Std. Error z value Pr**(>|**z**|)**

**(**Intercept**)** 4.489714 0.261171 17.191 **<**2e**-**16 *******

treat1 0.576422 0.035492 16.241 **<**2e**-**16 *******

treat2 0.007237 0.040089 0.181 0.8567

treat4 **-**0.073673 0.040922 **-**1.800 0.0718 .

treat5 **-**0.648171 0.048462 **-**13.375 **<**2e**-**16 *******

**---**

Signif. codes**:** 0 ‘*******’ 0.001 ‘******’ 0.01 ‘*****’ 0.05 ‘.’ 0.1 ‘ ’ 1

Correlation of Fixed Effects**:**

**(**Intr**)** treat1 treat2 treat4

treat1 **-**0.087

treat2 **-**0.077 0.567

treat4 **-**0.075 0.555 0.492

treat5 **-**0.064 0.469 0.415 0.407

**(**contrasts**(**bouts_data**$**treat**)** **<-** contr.treatment**(**levels**(**bouts_data**$**treat**)**,base**=**2**))**

modbouts1.2 **<-** glmer**(**bouts **~** treat **+** **(**1**|**colony**)**, data **=** bouts_data, family **=** 'poisson'**)**

summary**(**modbouts1.2**)**

**-----------------------------------------------------------------------------------------**

Generalized linear mixed model fit by maximum likelihood **(**Laplace

Approximation**)** **[**glmerMod**]**

Family**:** poisson **(** log **)**

Formula**:** bouts **~** treat **+** **(**1 **|** colony**)**

Data**:** bouts_data

AIC BIC logLik deviance df.resid

1282.5 1294.0 **-**635.3 1270.5 44

Scaled residuals**:**

Min 1Q Median 3Q Max

**-**7.9975 **-**2.7177 0.2405 2.0151 11.4490

Random effects**:**

Groups Name Variance Std.Dev.

colony **(**Intercept**)** 0.6727 0.8202

Number of obs**:** 50, groups**:** colony, 10

Fixed effects**:**

Estimate Std. Error z value Pr**(>|**z**|)**

**(**Intercept**)** 4.496935 0.261159 17.219 **<**2e**-**16 *******

treat1 0.569186 0.035410 16.074 **<**2e**-**16 *******

treat3 **-**0.007236 0.040089 **-**0.180 0.8568

treat4 **-**0.080910 0.040851 **-**1.981 0.0476 *****

treat5 **-**0.655404 0.048401 **-**13.541 **<**2e**-**16 *******

**---**

Signif. codes**:** 0 ‘*******’ 0.001 ‘******’ 0.01 ‘*****’ 0.05 ‘.’ 0.1 ‘ ’ 1

Correlation of Fixed Effects**:**

**(**Intr**)** treat1 treat3 treat4

treat1 **-**0.087

treat3 **-**0.076 0.564

treat4 **-**0.075 0.554 0.489

treat5 **-**0.063 0.467 0.413 0.405

1. Total path length analysis:

#Choosing best transformation

shapiro.test**(**bouts_data**$**tpl**)**

**-----------------------------------------------------**

Shapiro**-**Wilk normality test

data**:** bouts_data**$**tpl

W **=** 0.8948, p**-**value **=** 0.0003748

shapiro.test**(**log10**(**bouts_data**$**tpl **+** 0.1**))**

**-----------------------------------------------------**

Shapiro**-**Wilk normality test

data**:** log10**(**bouts_data**$**tpl **+** 0.1**)**

W **=** 0.9303, p**-**value **=** 0.006281

shapiro.test**(**sqrt**(**bouts_data**$**tpl**))**

**-----------------------------------------------------**

Shapiro**-**Wilk normality test

data**:** sqrt**(**bouts_data**$**tpl**)**

W **=** 0.981, p**-**value **=** 0.6072

##Models

**(**contrasts**(**bouts_data**$**treat**)** **<-** contr.poly**(**levels**((**bouts_data**$**treat**))))**

tplmod0 **<-** lmer**(**sqrt**(**tpl**)** **~** **(**1**|**colony**)**, data **=** bouts_data, REML **=** **FALSE)**

tplmod1 **<-** lmer**(**sqrt**(**tpl**)** **~** treat **+** **(**1**|**colony**)**, data **=** bouts_data, REML **=** **FALSE)**

summary**(**tplmod1**)**

**----------------------------------------------------------------------------------------**

Linear mixed model fit by maximum likelihood t**-**tests use Satterthwaite

approximations to degrees of freedom **[**merModLmerTest**]**

Formula**:** sqrt**(**tpl**)** **~** treat **+** **(**1 **|** colony**)**

Data**:** bouts_data

AIC BIC logLik deviance df.resid

577.3 590.7 **-**281.7 563.3 43

Scaled residuals**:**

Min 1Q Median 3Q Max

**-**2.29089 **-**0.44442 0.05647 0.47239 2.27932

Random effects**:**

Groups Name Variance Std.Dev.

colony **(**Intercept**)** 5104 71.44

Residual 2898 53.83

Number of obs**:** 50, groups**:** colony, 10

Fixed effects**:**

Estimate Std. Error df t value Pr**(>|**t**|)**

**(**Intercept**)** 205.35 23.84 10.00 8.614 6.13e**-**06 *******

treat.L **-**82.82 17.02 40.00 **-**4.865 1.82e**-**05 *******

treat.Q 0.99 17.02 40.00 0.058 0.95391

treat.C **-**48.55 17.02 40.00 **-**2.852 0.00684 ******

treat**^**4 **-**12.17 17.02 40.00 **-**0.715 0.47896

**---**

Signif. codes**:** 0 ‘*******’ 0.001 ‘******’ 0.01 ‘*****’ 0.05 ‘.’ 0.1 ‘ ’ 1

Correlation of Fixed Effects**:**

**(**Intr**)** tret.L tret.Q tret.C

treat.L 0.000

treat.Q 0.000 0.000

treat.C 0.000 0.000 0.000

treat**^**4 0.000 0.000 0.000 0.000

anova**(**tplmod0, tplmod1**)**

**----------------------------------------------------------------------------------------**

Data**:** bouts_data

Models**:**

object**:** sqrt**(**tpl**)** **~** **(**1 **|** colony**)**

..1**:** sqrt**(**tpl**)** **~** treat **+** **(**1 **|** colony**)**

Df AIC BIC logLik deviance Chisq Chi Df Pr**(>**Chisq**)**

object 3 593.00 598.74 **-**293.50 587.00

..1 7 577.31 590.70 **-**281.66 563.31 23.686 4 9.231e**-**05 *******

**---**

Signif. codes**:** 0 ‘*******’ 0.001 ‘******’ 0.01 ‘*****’ 0.05 ‘.’ 0.1 ‘ ’ 1

##Residuals

shapiro.test**(**resid**(**tplmod1**))**

**----------------------------------------------------------------------------------------**

Shapiro**-**Wilk normality test

data**:** resid**(**tplmod1**)**

W **=** 0.9842, p**-**value **=** 0.737

#Contrats

**(**contrasts**(**bouts_data**$**treat**)** **<-** contr.treatment**(**levels**(**bouts_data**$**treat**)**,base**=**5**))**

tplmod1.5 **<-** lmer**(**sqrt**(**tpl**)** **~** treat **+** **(**1**|**colony**)**, data **=** bouts_data, REML **=** **FALSE)**

summary**(**tplmod1.5**)**

**----------------------------------------------------------------------------------------**

Linear mixed model fit by maximum likelihood t**-**tests use Satterthwaite

approximations to degrees of freedom **[**merModLmerTest**]**

Formula**:** sqrt**(**tpl**)** **~** treat **+** **(**1 **|** colony**)**

Data**:** bouts_data

AIC BIC logLik deviance df.resid

577.3 590.7 **-**281.7 563.3 43

Scaled residuals**:**

Min 1Q Median 3Q Max

**-**2.29089 **-**0.44442 0.05647 0.47239 2.27932

Random effects**:**

Groups Name Variance Std.Dev.

colony **(**Intercept**)** 5104 71.44

Residual 2898 53.83

Number of obs**:** 50, groups**:** colony, 10

Fixed effects**:**

Estimate Std. Error df t value Pr**(>|**t**|)**

**(**Intercept**)** 136.69 28.29 19.03 4.832 0.000115 *******

treat1 135.46 24.08 40.00 5.627 1.58e**-**06 *******

treat2 69.69 24.08 40.00 2.895 0.006120 ******

treat3 59.40 24.08 40.00 2.467 0.017986 *****

treat4 78.73 24.08 40.00 3.270 0.002216 ******

**---**

Signif. codes**:** 0 ‘*******’ 0.001 ‘******’ 0.01 ‘*****’ 0.05 ‘.’ 0.1 ‘ ’ 1

Correlation of Fixed Effects**:**

**(**Intr**)** treat1 treat2 treat3

treat1 **-**0.426

treat2 **-**0.426 0.500

treat3 **-**0.426 0.500 0.500

treat4 **-**0.426 0.500 0.500 0.500

**(**contrasts**(**bouts_data**$**treat**)** **<-** contr.treatment**(**levels**(**bouts_data**$**treat**)**,base**=**4**))**

tplmod1.4 **<-** lmer**(**sqrt**(**tpl**)** **~** treat **+** **(**1**|**colony**)**, data **=** bouts_data, REML **=** **FALSE)**

summary**(**tplmod1.4**)**

**----------------------------------------------------------------------------------------**

Linear mixed model fit by maximum likelihood t**-**tests use Satterthwaite

approximations to degrees of freedom **[**merModLmerTest**]**

Formula**:** sqrt**(**tpl**)** **~** treat **+** **(**1 **|** colony**)**

Data**:** bouts_data

AIC BIC logLik deviance df.resid

577.3 590.7 **-**281.7 563.3 43

Scaled residuals**:**

Min 1Q Median 3Q Max

**-**2.29089 **-**0.44442 0.05647 0.47239 2.27932

Random effects**:**

Groups Name Variance Std.Dev.

colony **(**Intercept**)** 5104 71.44

Residual 2898 53.83

Number of obs**:** 50, groups**:** colony, 10

Fixed effects**:**

Estimate Std. Error df t value Pr**(>|**t**|)**

**(**Intercept**)** 215.422 28.288 19.030 7.615 3.42e**-**07 *******

treat1 56.736 24.075 40.000 2.357 0.02343 *****

treat2 **-**9.039 24.075 40.000 **-**0.375 0.70932

treat3 **-**19.325 24.075 40.000 **-**0.803 0.42688

treat5 **-**78.729 24.075 40.000 **-**3.270 0.00222 ******

**---**

Signif. codes**:** 0 ‘*******’ 0.001 ‘******’ 0.01 ‘*****’ 0.05 ‘.’ 0.1 ‘ ’ 1

Correlation of Fixed Effects**:**

**(**Intr**)** treat1 treat2 treat3

treat1 **-**0.426

treat2 **-**0.426 0.500

treat3 **-**0.426 0.500 0.500

treat5 **-**0.426 0.500 0.500 0.500

**(**contrasts**(**bouts_data**$**treat**)** **<-** contr.treatment**(**levels**(**bouts_data**$**treat**)**,base**=**3**))**

tplmod1.3 **<-** lmer**(**sqrt**(**tpl**)** **~** treat **+** **(**1**|**colony**)**, data **=** bouts_data, REML **=** **FALSE)**

summary**(**tplmod1.3**)**

**----------------------------------------------------------------------------------------**

Linear mixed model fit by maximum likelihood t**-**tests use Satterthwaite

approximations to degrees of freedom **[**merModLmerTest**]**

Formula**:** sqrt**(**tpl**)** **~** treat **+** **(**1 **|** colony**)**

Data**:** bouts_data

AIC BIC logLik deviance df.resid

577.3 590.7 **-**281.7 563.3 43

Scaled residuals**:**

Min 1Q Median 3Q Max

**-**2.29089 **-**0.44442 0.05647 0.47239 2.27932

Random effects**:**

Groups Name Variance Std.Dev.

colony **(**Intercept**)** 5104 71.44

Residual 2898 53.83

Number of obs**:** 50, groups**:** colony, 10

Fixed effects**:**

Estimate Std. Error df t value Pr**(>|**t**|)**

**(**Intercept**)** 196.10 28.29 19.03 6.932 1.3e**-**06 *******

treat1 76.06 24.08 40.00 3.159 0.00301 ******

treat2 10.29 24.08 40.00 0.427 0.67146

treat4 19.33 24.08 40.00 0.803 0.42688

treat5 **-**59.40 24.08 40.00 **-**2.467 0.01799 *****

**---**

Signif. codes**:** 0 ‘*******’ 0.001 ‘******’ 0.01 ‘*****’ 0.05 ‘.’ 0.1 ‘ ’ 1

Correlation of Fixed Effects**:**

**(**Intr**)** treat1 treat2 treat4

treat1 **-**0.426

treat2 **-**0.426 0.500

treat4 **-**0.426 0.500 0.500

treat5 **-**0.426 0.500 0.500 0.500

**(**contrasts**(**bouts_data**$**treat**)** **<-** contr.treatment**(**levels**(**bouts_data**$**treat**)**,base**=**2**))**

tplmod1.2 **<-** lmer**(**sqrt**(**tpl**)** **~** treat **+** **(**1**|**colony**)**, data **=** bouts_data, REML **=** **FALSE)**

summary**(**tplmod1.2**)**

**----------------------------------------------------------------------------------------**

Linear mixed model fit by maximum likelihood t**-**tests use Satterthwaite

approximations to degrees of freedom **[**merModLmerTest**]**

Formula**:** sqrt**(**tpl**)** **~** treat **+** **(**1 **|** colony**)**

Data**:** bouts_data

AIC BIC logLik deviance df.resid

577.3 590.7 **-**281.7 563.3 43

Scaled residuals**:**

Min 1Q Median 3Q Max

**-**2.29089 **-**0.44442 0.05647 0.47239 2.27932

Random effects**:**

Groups Name Variance Std.Dev.

colony **(**Intercept**)** 5104 71.44

Residual 2898 53.83

Number of obs**:** 50, groups**:** colony, 10

Fixed effects**:**

Estimate Std. Error df t value Pr**(>|**t**|)**

**(**Intercept**)** 206.384 28.288 19.030 7.296 6.34e**-**07 *******

treat1 65.775 24.075 40.000 2.732 0.00932 ******

treat3 **-**10.287 24.075 40.000 **-**0.427 0.67146

treat4 9.039 24.075 40.000 0.375 0.70932

treat5 **-**69.690 24.075 40.000 **-**2.895 0.00612 ******

**---**

Signif. codes**:** 0 ‘*******’ 0.001 ‘******’ 0.01 ‘*****’ 0.05 ‘.’ 0.1 ‘ ’ 1

Correlation of Fixed Effects**:**

**(**Intr**)** treat1 treat3 treat4

treat1 **-**0.426

treat3 **-**0.426 0.500

treat4 **-**0.426 0.500 0.500

treat5 **-**0.426 0.500 0.500 0.500

1. Total exploration time analysis:

#Choosing best transformation

shapiro.test**(**bouts_data**$**tet**)**

**-----------------------------------------------------**

Shapiro**-**Wilk normality test

data**:** bouts_data**$**tet

W **=** 0.925, p**-**value **=** 0.003603

shapiro.test**(**log10**(**bouts_data**$**tet **+** 0.1**))**

**-----------------------------------------------------**

Shapiro**-**Wilk normality test

data**:** log10**(**bouts_data**$**tet **+** 0.1**)**

W **=** 0.5439, p**-**value **=** 3.116e**-**11

shapiro.test**(**sqrt**(**bouts_data**$**tet**))**

**-----------------------------------------------------**

Shapiro**-**Wilk normality test

data**:** sqrt**(**bouts_data**$**tet**)**

W **=** 0.9879, p**-**value **=** 0.8865

##Models

**(**contrasts**(**bouts_data**$**treat**)** **<-** contr.poly**(**levels**((**bouts_data**$**treat**))))**

tetmod0 **<-** lmer**(**sqrt**(**tet**)** **~** **(**1**|**colony**)**, data **=** bouts_data, REML **=** **FALSE)**

tetmod1 **<-** lmer**(**sqrt**(**tet**)** **~** treat **+** **(**1**|**colony**)**, data **=** bouts_data, REML **=** **FALSE)**

summary**(**tetmod1**)**

**----------------------------------------------------------------------------------------**

Linear mixed model fit by maximum likelihood t**-**tests use Satterthwaite

approximations to degrees of freedom **[**merModLmerTest**]**

Formula**:** sqrt**(**tet**)** **~** treat **+** **(**1 **|** colony**)**

Data**:** bouts_data

AIC BIC logLik deviance df.resid

601.4 614.8 **-**293.7 587.4 43

Scaled residuals**:**

Min 1Q Median 3Q Max

**-**2.4577 **-**0.4753 0.1153 0.5632 2.3830

Random effects**:**

Groups Name Variance Std.Dev.

colony **(**Intercept**)** 6524 80.77

Residual 4939 70.28

Number of obs**:** 50, groups**:** colony, 10

Fixed effects**:**

Estimate Std. Error df t value Pr**(>|**t**|)**

**(**Intercept**)** 259.769 27.407 10.000 9.478 2.59e**-**06 *******

treat.L **-**87.499 22.223 40.000 **-**3.937 0.000321 *******

treat.Q **-**7.410 22.223 40.000 **-**0.333 0.740538

treat.C **-**44.796 22.223 40.000 **-**2.016 0.050576 .

treat**^**4 **-**8.602 22.223 40.000 **-**0.387 0.700750

**---**

Signif. codes**:** 0 ‘*******’ 0.001 ‘******’ 0.01 ‘*****’ 0.05 ‘.’ 0.1 ‘ ’ 1

Correlation of Fixed Effects**:**

**(**Intr**)** tret.L tret.Q tret.C

treat.L 0.000

treat.Q 0.000 0.000

treat.C 0.000 0.000 0.000

treat**^**4 0.000 0.000 0.000 0.000

anova**(**tetmod0, tetmod1**)**

**----------------------------------------------------------------------------------------**

Data**:** bouts_data

Models**:**

object**:** sqrt**(**tet**)** **~** **(**1 **|** colony**)**

..1**:** sqrt**(**tet**)** **~** treat **+** **(**1 **|** colony**)**

Df AIC BIC logLik deviance Chisq Chi Df Pr**(>**Chisq**)**

object 3 609.53 615.26 **-**301.76 603.53

..1 7 601.42 614.81 **-**293.71 587.42 16.103 4 0.002884 ******

**---**

Signif. codes**:** 0 ‘*******’ 0.001 ‘******’ 0.01 ‘*****’ 0.05 ‘.’ 0.1 ‘ ’ 1

#Residuals

shapiro.test**(**resid**(**tetmod1**))**

**----------------------------------------------------------------------------------------**

Shapiro**-**Wilk normality test

data**:** resid**(**tetmod1**)**

W **=** 0.9819, p**-**value **=** 0.6329

#Contrats

**(**contrasts**(**bouts_data**$**treat**)** **<-** contr.treatment**(**levels**(**bouts_data**$**treat**)**,base**=**5**))**

tetmod1.5 **<-** lmer**(**sqrt**(**tet**)** **~** treat **+** **(**1**|**colony**)**, data **=** bouts_data, REML **=** **FALSE)**

summary**(**tplmod1.5**)**

**----------------------------------------------------------------------------------------**

Linear mixed model fit by maximum likelihood t**-**tests use Satterthwaite

approximations to degrees of freedom **[**merModLmerTest**]**

Formula**:** sqrt**(**tpl**)** **~** treat **+** **(**1 **|** colony**)**

Data**:** bouts_data

AIC BIC logLik deviance df.resid

754.0 767.4 **-**370.0 740.0 43

Scaled residuals**:**

Min 1Q Median 3Q Max

**-**2.30723 **-**0.45115 0.05746 0.49680 2.26309

Random effects**:**

Groups Name Variance Std.Dev.

colony **(**Intercept**)** 182844 427.6

Residual 98334 313.6

Number of obs**:** 50, groups**:** colony, 10

Fixed effects**:**

Estimate Std. Error df t value Pr**(>|**t**|)**

**(**Intercept**)** 806.52 167.68 18.58 4.810 0.000129 *******

treat1 800.91 140.24 40.00 5.711 1.2e**-**06 *******

treat2 412.59 140.24 40.00 2.942 0.005401 ******

treat3 353.79 140.24 40.00 2.523 0.015723 *****

treat4 459.73 140.24 40.00 3.278 0.002167 ******

**---**

Signif. codes**:** 0 ‘*******’ 0.001 ‘******’ 0.01 ‘*****’ 0.05 ‘.’ 0.1 ‘ ’ 1

Correlation of Fixed Effects**:**

**(**Intr**)** treat1 treat2 treat3

treat1 **-**0.418

treat2 **-**0.418 0.500

treat3 **-**0.418 0.500 0.500

treat4 **-**0.418 0.500 0.500 0.500

**(**contrasts**(**bouts_data**$**treat**)** **<-** contr.treatment**(**levels**(**bouts_data**$**treat**)**,base**=**4**))**

tetmod1.4 **<-** lmer**(**sqrt**(**tet**)** **~** treat **+** **(**1**|**colony**)**, data **=** bouts_data, REML **=** **FALSE)**

summary**(**tplmod1.4**)**

**----------------------------------------------------------------------------------------**

Linear mixed model fit by maximum likelihood t**-**tests use Satterthwaite approximations to degrees of

freedom **[**merModLmerTest**]**

Formula**:** sqrt**(**tpl**)** **~** treat **+** **(**1 **|** colony**)**

Data**:** bouts_data

AIC BIC logLik deviance df.resid

754.0 767.4 **-**370.0 740.0 43

Scaled residuals**:**

Min 1Q Median 3Q Max

**-**2.30723 **-**0.45115 0.05746 0.49680 2.26309

Random effects**:**

Groups Name Variance Std.Dev.

colony **(**Intercept**)** 182844 427.6

Residual 98334 313.6

Number of obs**:** 50, groups**:** colony, 10

Fixed effects**:**

Estimate Std. Error df t value Pr**(>|**t**|)**

**(**Intercept**)** 1266.25 167.68 18.58 7.551 4.51e**-**07 *******

treat1 341.18 140.24 40.00 2.433 0.01955 *****

treat2 **-**47.14 140.24 40.00 **-**0.336 0.73853

treat3 **-**105.94 140.24 40.00 **-**0.755 0.45442

treat5 **-**459.73 140.24 40.00 **-**3.278 0.00217 ******

**---**

Signif. codes**:** 0 ‘*******’ 0.001 ‘******’ 0.01 ‘*****’ 0.05 ‘.’ 0.1 ‘ ’ 1

Correlation of Fixed Effects**:**

**(**Intr**)** treat1 treat2 treat3

treat1 **-**0.418

treat2 **-**0.418 0.500

treat3 **-**0.418 0.500 0.500

treat5 **-**0.418 0.500 0.500 0.500

**(**contrasts**(**bouts_data**$**treat**)** **<-** contr.treatment**(**levels**(**bouts_data**$**treat**)**,base**=**3**))**

tetmod1.3 **<-** lmer**(**sqrt**(**tet**)** **~** treat **+** **(**1**|**colony**)**, data **=** bouts_data, REML **=** **FALSE)**

summary**(**tplmod1.3**)**

**----------------------------------------------------------------------------------------**

Linear mixed model fit by maximum likelihood t**-**tests use Satterthwaite approximations to degrees of

freedom **[**merModLmerTest**]**

Formula**:** sqrt**(**tpl**)** **~** treat **+** **(**1 **|** colony**)**

Data**:** bouts_data

AIC BIC logLik deviance df.resid

754.0 767.4 **-**370.0 740.0 43

Scaled residuals**:**

Min 1Q Median 3Q Max

**-**2.30723 **-**0.45115 0.05746 0.49680 2.26309

Random effects**:**

Groups Name Variance Std.Dev.

colony **(**Intercept**)** 182844 427.6

Residual 98334 313.6

Number of obs**:** 50, groups**:** colony, 10

Fixed effects**:**

Estimate Std. Error df t value Pr**(>|**t**|)**

**(**Intercept**)** 1160.31 167.68 18.58 6.920 1.52e**-**06 *******

treat1 447.12 140.24 40.00 3.188 0.00278 ******

treat2 58.80 140.24 40.00 0.419 0.67725

treat4 105.94 140.24 40.00 0.755 0.45442

treat5 **-**353.79 140.24 40.00 **-**2.523 0.01572 *****

**---**

Signif. codes**:** 0 ‘*******’ 0.001 ‘******’ 0.01 ‘*****’ 0.05 ‘.’ 0.1 ‘ ’ 1

Correlation of Fixed Effects**:**

**(**Intr**)** treat1 treat2 treat4

treat1 **-**0.418

treat2 **-**0.418 0.500

treat4 **-**0.418 0.500 0.500

treat5 **-**0.418 0.500 0.500 0.500

**(**contrasts**(**bouts_data**$**treat**)** **<-** contr.treatment**(**levels**(**bouts_data**$**treat**)**,base**=**2**))**

tetmod1.2 **<-** lmer**(**sqrt**(**tet**)** **~** treat **+** **(**1**|**colony**)**, data **=** bouts_data, REML **=** **FALSE)**

summary**(**tplmod1.2**)**

**----------------------------------------------------------------------------------------**

Linear mixed model fit by maximum likelihood t**-**tests use Satterthwaite approximations to degrees of

freedom **[**merModLmerTest**]**

Formula**:** sqrt**(**tpl**)** **~** treat **+** **(**1 **|** colony**)**

Data**:** bouts_data

AIC BIC logLik deviance df.resid

754.0 767.4 **-**370.0 740.0 43

Scaled residuals**:**

Min 1Q Median 3Q Max

**-**2.30723 **-**0.45115 0.05746 0.49680 2.26309

Random effects**:**

Groups Name Variance Std.Dev.

colony **(**Intercept**)** 182844 427.6

Residual 98334 313.6

Number of obs**:** 50, groups**:** colony, 10

Fixed effects**:**

Estimate Std. Error df t value Pr**(>|**t**|)**

**(**Intercept**)** 1219.11 167.68 18.58 7.270 7.7e**-**07 *******

treat1 388.32 140.24 40.00 2.769 0.00848 ******

treat3 **-**58.80 140.24 40.00 **-**0.419 0.67725

treat4 47.14 140.24 40.00 0.336 0.73853

treat5 **-**412.59 140.24 40.00 **-**2.942 0.00540 ******

**---**

Signif. codes**:** 0 ‘*******’ 0.001 ‘******’ 0.01 ‘*****’ 0.05 ‘.’ 0.1 ‘ ’ 1

Correlation of Fixed Effects**:**

**(**Intr**)** treat1 treat3 treat4

treat1 **-**0.418

treat3 **-**0.418 0.500

treat4 **-**0.418 0.500 0.500

treat5 **-**0.418 0.500 0.500 0.500

1. Calculation of fitted values for Number of bouts, Total path length and Total exploration time:

##polynomial fitted lines

**(**contrasts**(**bouts_data**$**treat**)** **<-** contr.poly**(**levels**((**bouts_data**$**treat**))))**

.L .Q .C **^**4

**[**1,**]** **-**6.324555e**-**01 0.5345225 **-**3.162278e**-**01 0.1195229

**[**2,**]** **-**3.162278e**-**01 **-**0.2672612 6.324555e**-**01 **-**0.4780914

**[**3,**]** **-**3.287978e**-**17 **-**0.5345225 2.164914e**-**16 0.7171372

**[**4,**]** 3.162278e**-**01 **-**0.2672612 **-**6.324555e**-**01 **-**0.4780914

**[**5,**]** 6.324555e**-**01 0.5345225 3.162278e**-**01 0.1195229

##### Number of bouts

#Model output

L **=** **-**0.80009

Q **=** **-**0.02059

C **=** **-**0.33608

F **=** 0.02319

bout1 **=** 4.46207 **+** L***-**6.324555e**-**01 **+** Q*****0.5345225 **+** C***-**3.162278e**-**01 **+** F*****0.1195229

bout2 **=** 4.46207 **+** L***-**3.162278e**-**01 **+** Q***-**0.2672612 **+** C*****6.324555e**-**01 **+** F***-**0.4780914

bout3 **=** 4.46207 **+** L***-**3.287978e**-**17 **+** Q***-**0.5345225 **+** C*****2.164914e**-**16 **+** F*****0.7171372

bout4 **=** 4.46207 **+** L*****3.162278e**-**01 **+** Q***-**0.2672612 **+** C***-**6.324555e**-**01 **+** F***-**0.4780914

bout5 **=** 4.46207 **+** L*****6.324555e**-**01 **+** Q*****0.5345225 **+** C*****3.162278e**-**01 **+** F*****0.1195229

##### Total path length

#Model output

L **=** **-**447.85

Q **=** 42.86

C **=** **-**261.19

F **=** **-**59.33

pl1 **=** 1225.77 **+** L***-**6.324555e**-**01 **+** Q*****0.5345225 **+** C***-**3.162278e**-**01 **+** F*****0.1195229

pl2 **=** 1225.77 **+** L***-**3.162278e**-**01 **+** Q***-**0.2672612 **+** C*****6.324555e**-**01 **+** F***-**0.4780914

pl3 **=** 1225.77 **+** L***-**3.287978e**-**17 **+** Q***-**0.5345225 **+** C*****2.164914e**-**16 **+** F*****0.7171372

pl4 **=** 1225.77 **+** L*****3.162278e**-**01 **+** Q***-**0.2672612 **+** C***-**6.324555e**-**01 **+** F***-**0.4780914

pl5 **=** 1225.77 **+** L*****6.324555e**-**01 **+** Q*****0.5345225 **+** C*****3.162278e**-**01 **+** F*****0.1195229

##### Total exploration time

#Model output

L **=** **-**87.499

Q **=** **-**7.410

C **=** **-**44.796

F **=** **-**8.602

et1 **=** 259.769 **+** L***-**6.324555e**-**01 **+** Q*****0.5345225 **+** C***-**3.162278e**-**01 **+** F*****0.1195229

et2 **=** 259.769 **+** L***-**3.162278e**-**01 **+** Q***-**0.2672612 **+** C*****6.324555e**-**01 **+** F***-**0.4780914

et3 **=** 259.769 **+** L***-**3.287978e**-**17 **+** Q***-**0.5345225 **+** C*****2.164914e**-**16 **+** F*****0.7171372

et4 **=** 259.769 **+** L*****3.162278e**-**01 **+** Q***-**0.2672612 **+** C***-**6.324555e**-**01 **+** F***-**0.4780914

et5 **=** 259.769 **+** L*****6.324555e**-**01 **+** Q*****0.5345225 **+** C*****3.162278e**-**01 **+** F*****0.1195229

1. Bout path length analysis:

### Models

modpl0 **<-** lmer**(**log10**(**pl**)** **~** **(**1**|** colony**)**, REML **=** **FALSE**, data **=** my_data**)**

modpl1 **<-** lmer**(**log10**(**pl**)** **~** treat **+** **(**1**|** colony**)**, REML **=** **FALSE**, data **=** my_data**)**

summary**(**modpl1**)**

**-------------------------------------------------------------------------------------**

Linear mixed model fit by maximum likelihood t**-**tests use Satterthwaite

approximations to degrees of freedom **[**merModLmerTest**]**

Formula**:** log10**(**pl**)** **~** treat **+** **(**1 **|** colony**)**

Data**:** my_data

AIC BIC logLik deviance df.resid

13485.9 13533.3 **-**6735.9 13471.9 6484

Scaled residuals**:**

Min 1Q Median 3Q Max

**-**3.3889 **-**0.3753 0.2127 0.6925 2.2508

Random effects**:**

Groups Name Variance Std.Dev.

colony **(**Intercept**)** 0.02005 0.1416

Residual 0.46434 0.6814

Number of obs**:** 6491, groups**:** colony, 10

Fixed effects**:**

Estimate Std. Error df t value Pr**(>|**t**|)**

**(**Intercept**)** 2.308e**+**00 4.639e**-**02 1.000e**+**01 49.745 5.9e**-**13 *******

treat.L 5.577e**-**02 2.189e**-**02 6.480e**+**03 2.548 0.0108 *****

treat.Q **-**7.525e**-**03 2.082e**-**02 6.489e**+**03 **-**0.361 0.7178

treat.C **-**1.877e**-**02 2.025e**-**02 6.487e**+**03 **-**0.927 0.3540

treat**^**4 **-**1.979e**-**02 1.982e**-**02 6.490e**+**03 **-**0.999 0.3180

**---**

Signif. codes**:** 0 ‘*******’ 0.001 ‘******’ 0.01 ‘*****’ 0.05 ‘.’ 0.1 ‘ ’ 1

Correlation of Fixed Effects**:**

**(**Intr**)** tret.L tret.Q tret.C

treat.L 0.082

treat.Q 0.021 0.379

treat.C 0.031 0.074 0.219

treat**^**4 0.016 0.108 0.039 0.074

anova**(**modpl0, modpl1**)**

**-------------------------------------------------------------------------------------**

Data**:** my_data

Models**:**

object**:** log10**(**pl**)** **~** **(**1 **|** colony**)**

..1**:** log10**(**pl**)** **~** treat **+** **(**1 **|** colony**)**

Df AIC BIC logLik deviance Chisq Chi Df Pr**(>**Chisq**)**

object 3 13489 13509 **-**6741.3 13483

..1 7 13486 13533 **-**6735.9 13472 10.744 4 0.0296 *****

**---**

Signif. codes**:** 0 ‘*******’ 0.001 ‘******’ 0.01 ‘*****’ 0.05 ‘.’ 0.1 ‘ ’ 1

########Contrasts

**(**contrasts**(**my_data**$**treat**)** **<-** contr.treatment**(**levels**(**my_data**$**treat**)**,base**=**5**))**

modpl1.5 **<-**lmer**(**log10**(**pl**)** **~** treat **+** **(**1**|**colony**)**, REML **=** **FALSE**, data **=** my_data**)**

summary**(**modpl1.5**)**

**--------------------------------------------------------------------------------**

Linear mixed model fit by maximum likelihood t**-**tests use Satterthwaite

approximations to degrees of freedom **[**merModLmerTest**]**

Formula**:** log10**(**pl**)** **~** treat **+** **(**1 **|** colony**)**

Data**:** my_data

AIC BIC logLik deviance df.resid

13485.9 13533.3 **-**6735.9 13471.9 6484

Scaled residuals**:**

Min 1Q Median 3Q Max

**-**3.3889 **-**0.3753 0.2127 0.6925 2.2508

Random effects**:**

Groups Name Variance Std.Dev.

colony **(**Intercept**)** 0.02005 0.1416

Residual 0.46434 0.6814

Number of obs**:** 6491, groups**:** colony, 10

Fixed effects**:**

Estimate Std. Error df t value Pr**(>|**t**|)**

**(**Intercept**)** 2.33069 0.05325 17.00000 43.768 **<**2e**-**16 *******

treat1 **-**0.05867 0.03135 6489.00000 **-**1.872 0.0613 .

treat2 **-**0.04099 0.03384 6486.00000 **-**1.211 0.2258

treat3 **-**0.03312 0.03332 6488.00000 **-**0.994 0.3203

treat4 0.01803 0.03393 6488.00000 0.532 0.5951

**---**

Signif. codes**:** 0 ‘*******’ 0.001 ‘******’ 0.01 ‘*****’ 0.05 ‘.’ 0.1 ‘ ’ 1

Correlation of Fixed Effects**:**

**(**Intr**)** treat1 treat2 treat3

treat1 **-**0.463

treat2 **-**0.430 0.724

treat3 **-**0.417 0.709 0.657

treat4 **-**0.419 0.707 0.654 0.652

**(**contrasts**(**my_data**$**treat**)** **<-** contr.treatment**(**levels**(**my_data**$**treat**)**,base**=**4**))**

modpl1.4 **<-**lmer**(**log10**(**pl**)** **~** treat **+** **(**1**|**colony**)**, REML **=** **FALSE**, data **=** my_data**)**

summary**(**modpl1.4**)**

**--------------------------------------------------------------------------------**

Linear mixed model fit by maximum likelihood t**-**tests use Satterthwaite

approximations to degrees of freedom **[**merModLmerTest**]**

Formula**:** log10**(**pl**)** **~** treat **+** **(**1 **|** colony**)**

Data**:** my_data

AIC BIC logLik deviance df.resid

13485.9 13533.3 **-**6735.9 13471.9 6484

Scaled residuals**:**

Min 1Q Median 3Q Max

**-**3.3889 **-**0.3753 0.2127 0.6925 2.2508

Random effects**:**

Groups Name Variance Std.Dev.

colony **(**Intercept**)** 0.02005 0.1416

Residual 0.46434 0.6814

Number of obs**:** 6491, groups**:** colony, 10

Fixed effects**:**

Estimate Std. Error df t value Pr**(>|**t**|)**

**(**Intercept**)** 2.34873 0.04972 13.00000 47.240 1.11e**-**15 *******

treat1 **-**0.07671 0.02508 6491.00000 **-**3.059 0.00223 ******

treat2 **-**0.05902 0.02820 6491.00000 **-**2.093 0.03641 *****

treat3 **-**0.05115 0.02807 6486.00000 **-**1.822 0.06850 .

treat5 **-**0.01803 0.03393 6488.00000 **-**0.532 0.59509

**---**

Signif. codes**:** 0 ‘*******’ 0.001 ‘******’ 0.01 ‘*****’ 0.05 ‘.’ 0.1 ‘ ’ 1

Correlation of Fixed Effects**:**

**(**Intr**)** treat1 treat2 treat3

treat1 **-**0.332

treat2 **-**0.299 0.589

treat3 **-**0.285 0.572 0.512

treat5 **-**0.233 0.468 0.419 0.435

**(**contrasts**(**my_data**$**treat**)** **<-** contr.treatment**(**levels**(**my_data**$**treat**)**,base**=**3**))**

modpl1.3 **<-**lmer**(**log10**(**pl**)** **~** treat **+** **(**1**|**colony**)**, REML **=** **FALSE**, data **=** my_data**)**

summary**(**modpl1.3**)**

**--------------------------------------------------------------------------------**

Linear mixed model fit by maximum likelihood t**-**tests use Satterthwaite approximations to

degrees of freedom **[**merModLmerTest**]**

Formula**:** log10**(**pl**)** **~** treat **+** **(**1 **|** colony**)**

Data**:** my_data

AIC BIC logLik deviance df.resid

13485.9 13533.3 **-**6735.9 13471.9 6484

Scaled residuals**:**

Min 1Q Median 3Q Max

**-**3.3889 **-**0.3753 0.2127 0.6925 2.2508

Random effects**:**

Groups Name Variance Std.Dev.

colony **(**Intercept**)** 0.02005 0.1416

Residual 0.46434 0.6814

Number of obs**:** 6491, groups**:** colony, 10

Fixed effects**:**

Estimate Std. Error df t value Pr**(>|**t**|)**

**(**Intercept**)** 2.298e**+**00 4.966e**-**02 1.300e**+**01 46.269 1.78e**-**15 *******

treat1 **-**2.555e**-**02 2.472e**-**02 6.490e**+**03 **-**1.034 0.3013

treat2 **-**7.865e**-**03 2.780e**-**02 6.491e**+**03 **-**0.283 0.7773

treat4 5.115e**-**02 2.807e**-**02 6.486e**+**03 1.822 0.0685 .

treat5 3.312e**-**02 3.332e**-**02 6.488e**+**03 0.994 0.3203

**---**

Signif. codes**:** 0 ‘*******’ 0.001 ‘******’ 0.01 ‘*****’ 0.05 ‘.’ 0.1 ‘ ’ 1

Correlation of Fixed Effects**:**

**(**Intr**)** treat1 treat2 treat4

treat1 **-**0.327

treat2 **-**0.293 0.577

treat4 **-**0.280 0.555 0.491

treat5 **-**0.224 0.448 0.399 0.400

**(**contrasts**(**my_data**$**treat**)** **<-** contr.treatment**(**levels**(**my_data**$**treat**)**,base**=**2**))**

modpl1.2 **<-**lmer**(**log10**(**pl**)** **~** treat **+** **(**1**|**colony**)**, REML **=** **FALSE**, data **=** my_data**)**

summary**(**modpl1.2**)**

**--------------------------------------------------------------------------------**

Linear mixed model fit by maximum likelihood t**-**tests use Satterthwaite approximations to

degrees of freedom **[**merModLmerTest**]**

Formula**:** log10**(**pl**)** **~** treat **+** **(**1 **|** colony**)**

Data**:** my_data

AIC BIC logLik deviance df.resid

13485.9 13533.3 **-**6735.9 13471.9 6484

Scaled residuals**:**

Min 1Q Median 3Q Max

**-**3.3889 **-**0.3753 0.2127 0.6925 2.2508

Random effects**:**

Groups Name Variance Std.Dev.

colony **(**Intercept**)** 0.02005 0.1416

Residual 0.46434 0.6814

Number of obs**:** 6491, groups**:** colony, 10

Fixed effects**:**

Estimate Std. Error df t value Pr**(>|**t**|)**

**(**Intercept**)** 2.290e**+**00 4.929e**-**02 1.200e**+**01 46.455 3.55e**-**15 *******

treat1 **-**1.769e**-**02 2.432e**-**02 6.490e**+**03 **-**0.727 0.4670

treat3 7.865e**-**03 2.780e**-**02 6.491e**+**03 0.283 0.7773

treat4 5.902e**-**02 2.820e**-**02 6.491e**+**03 2.093 0.0364 *****

treat5 4.099e**-**02 3.384e**-**02 6.486e**+**03 1.211 0.2258

**---**

Signif. codes**:** 0 ‘*******’ 0.001 ‘******’ 0.01 ‘*****’ 0.05 ‘.’ 0.1 ‘ ’ 1

Correlation of Fixed Effects**:**

**(**Intr**)** treat1 treat3 treat4

treat1 **-**0.312

treat3 **-**0.269 0.557

treat4 **-**0.271 0.552 0.498

treat5 **-**0.221 0.458 0.429 0.413

1. Bout duration analysis:

### Models

modet0 **<-** lmer**(**log10**(**et**)** **~** **(**1**|**colony**)**,REML **=** **FALSE**, data **=** my_data**)**

modet1 **<-** lmer**(**log10**(**et**)** **~** treat **+** **(**1**|**colony**)**, REML **=** **FALSE**, data **=** my_data**)**

summary**(**modet1**)**

**--------------------------------------------------------------------------------**

Linear mixed model fit by maximum likelihood t**-**tests use Satterthwaite

approximations to degrees of freedom **[**merModLmerTest**]**

Formula**:** log10**(**et**)** **~** treat **+** **(**1 **|** colony**)**

Data**:** my_data

AIC BIC logLik deviance df.resid

12626.0 12673.4 **-**6306.0 12612.0 6484

Scaled residuals**:**

Min 1Q Median 3Q Max

**-**3.3298 **-**0.5191 0.1683 0.6987 2.9520

Random effects**:**

Groups Name Variance Std.Dev.

colony **(**Intercept**)** 0.03072 0.1753

Residual 0.40640 0.6375

Number of obs**:** 6491, groups**:** colony, 10

Fixed effects**:**

Estimate Std. Error df t value Pr**(>|**t**|)**

**(**Intercept**)** 2.52446 0.05659 10.00000 44.609 1.20e**-**12 *******

treat.L 0.10878 0.02049 6491.00000 5.310 1.13e**-**07 *******

treat.Q **-**0.01668 0.01948 6487.00000 **-**0.856 0.392

treat.C 0.01198 0.01895 6485.00000 0.632 0.527

treat**^**4 **-**0.02783 0.01855 6487.00000 **-**1.501 0.134

**---**

Signif. codes**:** 0 ‘*******’ 0.001 ‘******’ 0.01 ‘*****’ 0.05 ‘.’ 0.1 ‘ ’ 1

Correlation of Fixed Effects**:**

**(**Intr**)** tret.L tret.Q tret.C

treat.L 0.063

treat.Q 0.016 0.379

treat.C 0.024 0.074 0.219

treat**^**4 0.013 0.109 0.039 0.074

anova**(**modet0, modet1**)**

**--------------------------------------------------------------------------------**

Data**:** my_data

Models**:**

object**:** log10**(**et**)** **~** **(**1 **|** colony**)**

..1**:** log10**(**et**)** **~** treat **+** **(**1 **|** colony**)**

Df AIC BIC logLik deviance Chisq Chi Df Pr**(>**Chisq**)**

object 3 12661 12682 **-**6327.6 12655

..1 7 12626 12673 **-**6306.0 12612 43.146 4 9.651e**-**09 *******

**---**

Signif. codes**:** 0 ‘*******’ 0.001 ‘******’ 0.01 ‘*****’ 0.05 ‘.’ 0.1 ‘ ’ 1

########Contrasts

**(**contrasts**(**my_data**$**treat**)** **<-** contr.treatment**(**levels**(**my_data**$**treat**)**,base**=**5**))**

modet1.5 **<-**lmer**(**log10**(**et**)** **~** treat **+** **(**1**|**colony**)**, REML **=** **FALSE**, data **=** my_data**)**

summary**(**modet1.5**)**

**--------------------------------------------------------------------------------**

Linear mixed model fit by maximum likelihood t**-**tests use Satterthwaite approximations to degrees of

freedom **[**merModLmerTest**]**

Formula**:** log10**(**et**)** **~** treat **+** **(**1 **|** colony**)**

Data**:** my_data

AIC BIC logLik deviance df.resid

12626.0 12673.4 **-**6306.0 12612.0 6484

Scaled residuals**:**

Min 1Q Median 3Q Max

**-**3.3298 **-**0.5191 0.1683 0.6987 2.9520

Random effects**:**

Groups Name Variance Std.Dev.

colony **(**Intercept**)** 0.03072 0.1753

Residual 0.40640 0.6375

Number of obs**:** 6491, groups**:** colony, 10

Fixed effects**:**

Estimate Std. Error df t value Pr**(>|**t**|)**

**(**Intercept**)** 2.58480 0.06165 14.00000 41.924 6.66e**-**16 *******

treat1 **-**0.14518 0.02934 6491.00000 **-**4.948 7.70e**-**07 *******

treat2 **-**0.06940 0.03167 6491.00000 **-**2.191 0.0285 *****

treat3 **-**0.07138 0.03118 6486.00000 **-**2.289 0.0221 *****

treat4 **-**0.01576 0.03175 6485.00000 **-**0.496 0.6196

**---**

Signif. codes**:** 0 ‘*******’ 0.001 ‘******’ 0.01 ‘*****’ 0.05 ‘.’ 0.1 ‘ ’ 1

Correlation of Fixed Effects**:**

**(**Intr**)** treat1 treat2 treat3

treat1 **-**0.374

treat2 **-**0.348 0.724

treat3 **-**0.337 0.709 0.657

treat4 **-**0.339 0.707 0.654 0.652

**(**contrasts**(**my_data**$**treat**)** **<-** contr.treatment**(**levels**(**my_data**$**treat**)**,base**=**4**))**

modet1.4 **<-**lmer**(**log10**(**et**)** **~** treat **+** **(**1**|**colony**)**, REML **=** **FALSE**, data **=** my_data**)**

summary**(**modet1.4**)**

**--------------------------------------------------------------------------------**

Linear mixed model fit by maximum likelihood t**-**tests use Satterthwaite approximations to degrees of

freedom **[**merModLmerTest**]**

Formula**:** log10**(**et**)** **~** treat **+** **(**1 **|** colony**)**

Data**:** my_data

AIC BIC logLik deviance df.resid

12626.0 12673.4 **-**6306.0 12612.0 6484

Scaled residuals**:**

Min 1Q Median 3Q Max

**-**3.3298 **-**0.5191 0.1683 0.6987 2.9520

Random effects**:**

Groups Name Variance Std.Dev.

colony **(**Intercept**)** 0.03072 0.1753

Residual 0.40640 0.6375

Number of obs**:** 6491, groups**:** colony, 10

Fixed effects**:**

Estimate Std. Error df t value Pr**(>|**t**|)**

**(**Intercept**)** 2.56904 0.05901 12.00000 43.533 3.31e**-**14 *******

treat1 **-**0.12942 0.02347 6489.00000 **-**5.515 3.63e**-**08 *******

treat2 **-**0.05364 0.02639 6490.00000 **-**2.032 0.0422 *****

treat3 **-**0.05562 0.02627 6484.00000 **-**2.118 0.0343 *****

treat5 0.01576 0.03175 6485.00000 0.496 0.6196

**---**

Signif. codes**:** 0 ‘*******’ 0.001 ‘******’ 0.01 ‘*****’ 0.05 ‘.’ 0.1 ‘ ’ 1

Correlation of Fixed Effects**:**

**(**Intr**)** treat1 treat2 treat3

treat1 **-**0.262

treat2 **-**0.236 0.589

treat3 **-**0.224 0.572 0.512

treat5 **-**0.184 0.468 0.418 0.435

**(**contrasts**(**my_data**$**treat**)** **<-** contr.treatment**(**levels**(**my_data**$**treat**)**,base**=**3**))**

modet1.3 **<-**lmer**(**log10**(**et**)** **~** treat **+** **(**1**|**colony**)**, REML **=** **FALSE**, data **=** my_data**)**

summary**(**modet1.3**)**

**--------------------------------------------------------------------------------**

Linear mixed model fit by maximum likelihood t**-**tests use Satterthwaite approximations to degrees of

freedom **[**merModLmerTest**]**

Formula**:** log10**(**et**)** **~** treat **+** **(**1 **|** colony**)**

Data**:** my_data

AIC BIC logLik deviance df.resid

12626.0 12673.4 **-**6306.0 12612.0 6484

Scaled residuals**:**

Min 1Q Median 3Q Max

**-**3.3298 **-**0.5191 0.1683 0.6987 2.9520

Random effects**:**

Groups Name Variance Std.Dev.

colony **(**Intercept**)** 0.03072 0.1753

Residual 0.40640 0.6375

Number of obs**:** 6491, groups**:** colony, 10

Fixed effects**:**

Estimate Std. Error df t value Pr**(>|**t**|)**

**(**Intercept**)** 2.513e**+**00 5.897e**-**02 1.200e**+**01 42.623 4.55e**-**14 *******

treat1 **-**7.379e**-**02 2.314e**-**02 6.491e**+**03 **-**3.189 0.00143 ******

treat2 1.982e**-**03 2.602e**-**02 6.490e**+**03 0.076 0.93928

treat4 5.562e**-**02 2.627e**-**02 6.484e**+**03 2.118 0.03425 *****

treat5 7.138e**-**02 3.118e**-**02 6.486e**+**03 2.289 0.02209 *****

**---**

Signif. codes**:** 0 ‘*******’ 0.001 ‘******’ 0.01 ‘*****’ 0.05 ‘.’ 0.1 ‘ ’ 1

Correlation of Fixed Effects**:**

**(**Intr**)** treat1 treat2 treat4

treat1 **-**0.258

treat2 **-**0.231 0.577

treat4 **-**0.221 0.555 0.490

treat5 **-**0.176 0.448 0.398 0.400

**(**contrasts**(**my_data**$**treat**)** **<-** contr.treatment**(**levels**(**my_data**$**treat**)**,base**=**2**))**

modet1.2 **<-**lmer**(**log10**(**et**)** **~** treat **+** **(**1**|**colony**)**, REML **=** **FALSE**, data **=** my_data**)**

summary**(**modet1.2**)**

**--------------------------------------------------------------------------------**

Linear mixed model fit by maximum likelihood t**-**tests use Satterthwaite approximations to degrees of

freedom **[**merModLmerTest**]**

Formula**:** log10**(**et**)** **~** treat **+** **(**1 **|** colony**)**

Data**:** my_data

AIC BIC logLik deviance df.resid

12626.0 12673.4 **-**6306.0 12612.0 6484

Scaled residuals**:**

Min 1Q Median 3Q Max

**-**3.3298 **-**0.5191 0.1683 0.6987 2.9520

Random effects**:**

Groups Name Variance Std.Dev.

colony **(**Intercept**)** 0.03072 0.1753

Residual 0.40640 0.6375

Number of obs**:** 6491, groups**:** colony, 10

Fixed effects**:**

Estimate Std. Error df t value Pr**(>|**t**|)**

**(**Intercept**)** 2.515e**+**00 5.870e**-**02 1.100e**+**01 42.855 6.64e**-**14 *******

treat1 **-**7.578e**-**02 2.275e**-**02 6.488e**+**03 **-**3.330 0.000873 *******

treat3 **-**1.982e**-**03 2.602e**-**02 6.490e**+**03 **-**0.076 0.939281

treat4 5.364e**-**02 2.639e**-**02 6.490e**+**03 2.032 0.042154 *****

treat5 6.940e**-**02 3.167e**-**02 6.491e**+**03 2.191 0.028466 *****

**---**

Signif. codes**:** 0 ‘*******’ 0.001 ‘******’ 0.01 ‘*****’ 0.05 ‘.’ 0.1 ‘ ’ 1

Correlation of Fixed Effects**:**

**(**Intr**)** treat1 treat3 treat4

treat1 **-**0.245

treat3 **-**0.211 0.557

treat4 **-**0.213 0.552 0.498

treat5 **-**0.174 0.458 0.430 0.414

1. Bout instantaneous speed analysis:

### Models

modsp0 **<-** lmer**(**sp **~** **(**1**|**colony**)**, REML **=** **FALSE**, data **=** my_data**)**

modsp1 **<-** lmer**(**sp **~** treat **+** **(**1**|**colony**)**, REML **=** **FALSE**, data **=** my_data**)**

Linear mixed model fit by maximum likelihood t**-**tests use Satterthwaite approximations to

degrees of freedom **[**merModLmerTest**]**

Formula**:** sp **~** treat **+** **(**1 **|** colony**)**

Data**:** my_data

AIC BIC logLik deviance df.resid

2905.6 2953.0 **-**1445.8 2891.6 6484

Scaled residuals**:**

Min 1Q Median 3Q Max

**-**2.9586 **-**0.6226 **-**0.0228 0.5826 3.8532

Random effects**:**

Groups Name Variance Std.Dev.

colony **(**Intercept**)** 0.01003 0.1002

Residual 0.09085 0.3014

Number of obs**:** 6491, groups**:** colony, 10

Fixed effects**:**

Estimate Std. Error df t value Pr**(>|**t**|)**

**(**Intercept**)** 6.572e**-**01 3.213e**-**02 1.000e**+**01 20.454 1.73e**-**09 *******

treat.L **-**9.054e**-**02 9.690e**-**03 6.491e**+**03 **-**9.344 **<** 2e**-**16 *******

treat.Q 1.360e**-**02 9.213e**-**03 6.485e**+**03 1.476 0.140

treat.C **-**4.896e**-**02 8.960e**-**03 6.484e**+**03 **-**5.464 4.82e**-**08 *******

treat**^**4 1.188e**-**02 8.769e**-**03 6.485e**+**03 1.354 0.176

**---**

Signif. codes**:** 0 ‘*******’ 0.001 ‘******’ 0.01 ‘*****’ 0.05 ‘.’ 0.1 ‘ ’ 1

Correlation of Fixed Effects**:**

**(**Intr**)** tret.L tret.Q tret.C

treat.L 0.052

treat.Q 0.013 0.379

treat.C 0.020 0.074 0.219

treat**^**4 0.011 0.109 0.039 0.074

anova**(**modsp0, modsp1**)**

**--------------------------------------------------------------------------------**

Data**:** my_data

Models**:**

object**:** sp **~** **(**1 **|** colony**)**

..1**:** sp **~** treat **+** **(**1 **|** colony**)**

Df AIC BIC logLik deviance Chisq Chi Df Pr**(>**Chisq**)**

object 3 3056.8 3077.2 **-**1525.4 3050.8

..1 7 2905.6 2953.0 **-**1445.8 2891.6 159.23 4 **<** 2.2e**-**16 *******

**---**

Signif. codes**:** 0 ‘*******’ 0.001 ‘******’ 0.01 ‘*****’ 0.05 ‘.’ 0.1 ‘ ’ 1

###Contrats

**(**contrasts**(**my_data**$**treat**)** **<-** contr.treatment**(**levels**(**my_data**$**treat**)**,base**=**5**))**

modsp1.5 **<-** lmer**(**sp **~** treat **+** **(**1**|**colony**)**, REML **=** **FALSE**, data **=** my_data**)**

summary**(**modsp1.5**)**

**--------------------------------------------------------------------------------**

Linear mixed model fit by maximum likelihood t**-**tests use Satterthwaite approximations to

degrees of freedom **[**merModLmerTest**]**

Formula**:** sp **~** treat **+** **(**1 **|** colony**)**

Data**:** my_data

AIC BIC logLik deviance df.resid

2905.6 2953.0 **-**1445.8 2891.6 6484

Scaled residuals**:**

Min 1Q Median 3Q Max

**-**2.9586 **-**0.6226 **-**0.0228 0.5826 3.8532

Random effects**:**

Groups Name Variance Std.Dev.

colony **(**Intercept**)** 0.01003 0.1002

Residual 0.09085 0.3014

Number of obs**:** 6491, groups**:** colony, 10

Fixed effects**:**

Estimate Std. Error df t value Pr**(>|**t**|)**

**(**Intercept**)** 5.932e**-**01 3.415e**-**02 1.300e**+**01 17.369 2.97e**-**10 *******

treat1 1.455e**-**01 1.388e**-**02 6.489e**+**03 10.484 **<** 2e**-**16 *******

treat2 5.241e**-**02 1.498e**-**02 6.490e**+**03 3.499 0.000470 *******

treat3 6.530e**-**02 1.474e**-**02 6.485e**+**03 4.429 9.62e**-**06 *******

treat4 5.708e**-**02 1.501e**-**02 6.484e**+**03 3.802 0.000145 *******

**---**

Signif. codes**:** 0 ‘*******’ 0.001 ‘******’ 0.01 ‘*****’ 0.05 ‘.’ 0.1 ‘ ’ 1

Correlation of Fixed Effects**:**

**(**Intr**)** treat1 treat2 treat3

treat1 **-**0.319

treat2 **-**0.297 0.724

treat3 **-**0.288 0.709 0.657

treat4 **-**0.289 0.707 0.654 0.652

**(**contrasts**(**my_data**$**treat**)** **<-** contr.treatment**(**levels**(**my_data**$**treat**)**,base**=**4**))**

modsp1.4 **<-** lmer**(**sp **~** treat **+** **(**1**|**colony**)**, REML **=** **FALSE**, data **=** my_data**)**

summary**(**modsp1.4**)**

**--------------------------------------------------------------------------------**

Linear mixed model fit by maximum likelihood t**-**tests use Satterthwaite approximations to

degrees of freedom **[**merModLmerTest**]**

Formula**:** sp **~** treat **+** **(**1 **|** colony**)**

Data**:** my_data

AIC BIC logLik deviance df.resid

2905.6 2953.0 **-**1445.8 2891.6 6484

Scaled residuals**:**

Min 1Q Median 3Q Max

**-**2.9586 **-**0.6226 **-**0.0228 0.5826 3.8532

Random effects**:**

Groups Name Variance Std.Dev.

colony **(**Intercept**)** 0.01003 0.1002

Residual 0.09085 0.3014

Number of obs**:** 6491, groups**:** colony, 10

Fixed effects**:**

Estimate Std. Error df t value Pr**(>|**t**|)**

**(**Intercept**)** 6.503e**-**01 3.309e**-**02 1.100e**+**01 19.650 4.65e**-**10 *******

treat1 8.841e**-**02 1.110e**-**02 6.487e**+**03 7.967 2.00e**-**15 *******

treat2 **-**4.669e**-**03 1.248e**-**02 6.488e**+**03 **-**0.374 0.708313

treat3 8.223e**-**03 1.242e**-**02 6.484e**+**03 0.662 0.507966

treat5 **-**5.708e**-**02 1.501e**-**02 6.484e**+**03 **-**3.802 0.000145 *******

**---**

Signif. codes**:** 0 ‘*******’ 0.001 ‘******’ 0.01 ‘*****’ 0.05 ‘.’ 0.1 ‘ ’ 1

Correlation of Fixed Effects**:**

**(**Intr**)** treat1 treat2 treat3

treat1 **-**0.221

treat2 **-**0.199 0.589

treat3 **-**0.189 0.572 0.512

treat5 **-**0.155 0.468 0.418 0.435

**(**contrasts**(**my_data**$**treat**)** **<-** contr.treatment**(**levels**(**my_data**$**treat**)**,base**=**3**))**

modsp1.3 **<-** lmer**(**sp **~** treat **+** **(**1**|**colony**)**, REML **=** **FALSE**, data **=** my_data**)**

summary**(**modsp1.3**)**

**--------------------------------------------------------------------------------**

Linear mixed model fit by maximum likelihood t**-**tests use Satterthwaite approximations to

degrees of freedom **[**merModLmerTest**]**

Formula**:** sp **~** treat **+** **(**1 **|** colony**)**

Data**:** my_data

AIC BIC logLik deviance df.resid

2905.6 2953.0 **-**1445.8 2891.6 6484

Scaled residuals**:**

Min 1Q Median 3Q Max

**-**2.9586 **-**0.6226 **-**0.0228 0.5826 3.8532

Random effects**:**

Groups Name Variance Std.Dev.

colony **(**Intercept**)** 0.01003 0.1002

Residual 0.09085 0.3014

Number of obs**:** 6491, groups**:** colony, 10

Fixed effects**:**

Estimate Std. Error df t value Pr**(>|**t**|)**

**(**Intercept**)** 6.585e**-**01 3.308e**-**02 1.100e**+**01 19.909 4.16e**-**10 *******

treat1 8.019e**-**02 1.094e**-**02 6.489e**+**03 7.329 2.61e**-**13 *******

treat2 **-**1.289e**-**02 1.230e**-**02 6.488e**+**03 **-**1.048 0.295

treat4 **-**8.223e**-**03 1.242e**-**02 6.484e**+**03 **-**0.662 0.508

treat5 **-**6.530e**-**02 1.474e**-**02 6.485e**+**03 **-**4.429 9.62e**-**06 *******

**---**

Signif. codes**:** 0 ‘*******’ 0.001 ‘******’ 0.01 ‘*****’ 0.05 ‘.’ 0.1 ‘ ’ 1

Correlation of Fixed Effects**:**

**(**Intr**)** treat1 treat2 treat4

treat1 **-**0.218

treat2 **-**0.195 0.577

treat4 **-**0.186 0.555 0.490

treat5 **-**0.149 0.448 0.398 0.400

**(**contrasts**(**my_data**$**treat**)** **<-** contr.treatment**(**levels**(**my_data**$**treat**)**,base**=**2**))**

modsp1.2 **<-** lmer**(**sp **~** treat **+** **(**1**|**colony**)**, REML **=** **FALSE**, data **=** my_data**)**

summary**(**modsp1.2**)**

**--------------------------------------------------------------------------------**

Linear mixed model fit by maximum likelihood t**-**tests use Satterthwaite approximations to

degrees of freedom **[**merModLmerTest**]**

Formula**:** sp **~** treat **+** **(**1 **|** colony**)**

Data**:** my_data

AIC BIC logLik deviance df.resid

2905.6 2953.0 **-**1445.8 2891.6 6484

Scaled residuals**:**

Min 1Q Median 3Q Max

**-**2.9586 **-**0.6226 **-**0.0228 0.5826 3.8532

Random effects**:**

Groups Name Variance Std.Dev.

colony **(**Intercept**)** 0.01003 0.1002

Residual 0.09085 0.3014

Number of obs**:** 6491, groups**:** colony, 10

Fixed effects**:**

Estimate Std. Error df t value Pr**(>|**t**|)**

**(**Intercept**)** 6.456e**-**01 3.297e**-**02 1.100e**+**01 19.584 6.06e**-**10 *******

treat1 9.308e**-**02 1.076e**-**02 6.486e**+**03 8.651 **<** 2e**-**16 *******

treat3 1.289e**-**02 1.230e**-**02 6.488e**+**03 1.048 0.29479

treat4 4.669e**-**03 1.248e**-**02 6.488e**+**03 0.374 0.70831

treat5 **-**5.241e**-**02 1.498e**-**02 6.490e**+**03 **-**3.499 0.00047 *******

**---**

Signif. codes**:** 0 ‘*******’ 0.001 ‘******’ 0.01 ‘*****’ 0.05 ‘.’ 0.1 ‘ ’ 1

Correlation of Fixed Effects**:**

**(**Intr**)** treat1 treat3 treat4

treat1 **-**0.206

treat3 **-**0.178 0.557

treat4 **-**0.179 0.552 0.498

treat5 **-**0.147 0.458 0.430 0.414

1. Calculation of fitted values for Bout path length, duration and instantaneous speed:

##polynomial fitted lines

**(**contrasts**(**my_data**$**treat**)** **<-** contr.poly**(**levels**((**my_data**$**treat**))))**

.L .Q .C **^**4

**[**1,**]** **-**6.324555e**-**01 0.5345225 **-**3.162278e**-**01 0.1195229

**[**2,**]** **-**3.162278e**-**01 **-**0.2672612 6.324555e**-**01 **-**0.4780914

**[**3,**]** **-**3.287978e**-**17 **-**0.5345225 2.164914e**-**16 0.7171372

**[**4,**]** 3.162278e**-**01 **-**0.2672612 **-**6.324555e**-**01 **-**0.4780914

**[**5,**]** 6.324555e**-**01 0.5345225 3.162278e**-**01 0.1195229

##### Bout path length

#Model output

L **=** 5.577e**-**02

Q **=** **-**7.525e**-**03

C **=** **-**1.877e**-**02

F **=** **-**1.979e**-**02

pl1 **=** 2.308e**+**00 **+** L***-**6.324555e**-**01 **+** Q*****0.5345225 **+** C***-**3.162278e**-**01 **+** F*****0.1195229

pl2 **=** 2.308e**+**00 **+** L***-**3.162278e**-**01 **+** Q***-**0.2672612 **+** C*****6.324555e**-**01 **+** F***-**0.4780914

pl3 **=** 2.308e**+**00 **+** L***-**3.287978e**-**17 **+** Q***-**0.5345225 **+** C*****2.164914e**-**16 **+** F*****0.7171372

pl4 **=** 2.308e**+**00 **+** L*****3.162278e**-**01 **+** Q***-**0.2672612 **+** C***-**6.324555e**-**01 **+** F***-**0.4780914

pl5 **=** 2.308e**+**00 **+** L*****6.324555e**-**01 **+** Q*****0.5345225 **+** C*****3.162278e**-**01 **+** F*****0.1195229

##### Bout duration

#Model output

L **=** 0.10878

Q **=** **-**0.01668

C **=** 0.01198

F **=** **-**0.02783

et1 **=** 2.52446 **+** L***-**6.324555e**-**01 **+** Q*****0.5345225 **+** C***-**3.162278e**-**01 **+** F*****0.1195229

et2 **=** 2.52446 **+** L***-**3.162278e**-**01 **+** Q***-**0.2672612 **+** C*****6.324555e**-**01 **+** F***-**0.4780914

et3 **=** 2.52446 **+** L***-**3.287978e**-**17 **+** Q***-**0.5345225 **+** C*****2.164914e**-**16 **+** F*****0.7171372

et4 **=** 2.52446 **+** L*****3.162278e**-**01 **+** Q***-**0.2672612 **+** C***-**6.324555e**-**01 **+** F***-**0.4780914

et5 **=** 2.52446 **+** L*****6.324555e**-**01 **+** Q*****0.5345225 **+** C*****3.162278e**-**01 **+** F*****0.1195229

#### Instantaneous speed

#Model output

L **=** **-**9.054e**-**02

Q **=** 1.360e**-**02

C **=** **-**4.896e**-**02

F **=** 1.188e**-**02

sp1 **=** 6.572e**-**01 **+** L***-**6.324555e**-**01 **+** Q*****0.5345225 **+** C***-**3.162278e**-**01 **+** F*****0.1195229

sp2 **=** 6.572e**-**01 **+** L***-**3.162278e**-**01 **+** Q***-**0.2672612 **+** C*****6.324555e**-**01 **+** F***-**0.4780914

sp3 **=** 6.572e**-**01 **+** L***-**3.287978e**-**17 **+** Q***-**0.5345225 **+** C*****2.164914e**-**16 **+** F*****0.7171372

sp4 **=** 6.572e**-**01 **+** L*****3.162278e**-**01 **+** Q***-**0.2672612 **+** C***-**6.324555e**-**01 **+** F***-**0.4780914

sp5 **=** 6.572e**-**01 **+** L*****6.324555e**-**01 **+** Q*****0.5345225 **+** C*****3.162278e**-**01 **+** F*****0.1195229

1. Total path length and total exploration time divided by number of bouts analysis:

#Total path length

tplmod2 **<-** lmer**(**sqrt**(**tpl**/**bouts**)** **~** treat **+** **(**1**|**colony**)**, data **=** bouts_data, REML **=** **FALSE)**

summary**(**tplmod2**)**

**---------------------------------------------------------------------------------**

Linear mixed model fit by maximum likelihood t**-**tests use Satterthwaite

approximations to degrees of freedom **[**merModLmerTest**]**

Formula**:** sqrt**(**tpl**/**bouts**)** **~** treat **+** **(**1 **|** colony**)**

Data**:** bouts_data

AIC BIC logLik deviance df.resid

292.2 305.4 **-**139.1 278.2 42

Scaled residuals**:**

Min 1Q Median 3Q Max

**-**2.48836 **-**0.36019 **-**0.06256 0.40610 2.86696

Random effects**:**

Groups Name Variance Std.Dev.

colony **(**Intercept**)** 10.56 3.250

Residual 12.19 3.492

Number of obs**:** 49, groups**:** colony, 10

Fixed effects**:**

Estimate Std. Error df t value Pr**(>|**t**|)**

**(**Intercept**)** 21.7479 1.1431 10.0700 19.025 3.16e**-**09 *******

treat.L 2.9721 1.1331 39.2400 2.623 0.0123 *****

treat.Q 1.1026 1.1249 39.2000 0.980 0.3330

treat.C **-**0.3528 1.1115 39.1100 **-**0.317 0.7526

treat**^**4 0.1246 1.1052 39.0700 0.113 0.9108

**---**

Signif. codes**:** 0 ‘*******’ 0.001 ‘******’ 0.01 ‘*****’ 0.05 ‘.’ 0.1 ‘ ’ 1

Correlation of Fixed Effects**:**

**(**Intr**)** tret.L tret.Q tret.C

treat.L 0.016

treat.Q 0.013 0.043

treat.C 0.008 0.026 0.022

treat**^**4 0.003 0.010 0.008 0.005

anova**(**tplmod0, tplmod2**)**

**---------------------------------------------------------------------------**

Data**:** bouts_data

Models**:**

object**:** sqrt**(**tpl**)** **~** **(**1 **|** colony**)**

..1**:** sqrt**(**tpl**/**bouts**)** **~** treat **+** **(**1 **|** colony**)**

Df AIC BIC logLik deviance Chisq Chi Df Pr**(>**Chisq**)**

object 3 593.00 598.74 **-**293.50 587.00

..1 7 292.15 305.40 **-**139.08 278.15 308.85 4 **<** 2.2e**-**16 *******

**---**

Signif. codes**:** 0 ‘*******’ 0.001 ‘******’ 0.01 ‘*****’ 0.05 ‘.’ 0.1 ‘ ’ 1

#Total exploration duration

tetmod2 **<-** lmer**(**sqrt**(**tet**/**bouts**)** **~** treat **+** **(**1**|**colony**)**, data **=** bouts_data, REML **=** **FALSE)**

summary**(**tetmod2**)**

**---------------------------------------------------------------------------**

Linear mixed model fit by maximum likelihood t**-**tests use Satterthwaite

approximations to degrees of freedom **[**merModLmerTest**]**

Formula**:** sqrt**(**tet**/**bouts**)** **~** treat **+** **(**1 **|** colony**)**

Data**:** bouts_data

AIC BIC logLik deviance df.resid

336.1 349.4 **-**161.1 322.1 42

Scaled residuals**:**

Min 1Q Median 3Q Max

**-**2.06027 **-**0.32761 **-**0.07792 0.21692 2.99688

Random effects**:**

Groups Name Variance Std.Dev.

colony **(**Intercept**)** 27.01 5.197

Residual 29.68 5.447

Number of obs**:** 49, groups**:** colony, 10

Fixed effects**:**

Estimate Std. Error df t value Pr**(>|**t**|)**

**(**Intercept**)** 28.436 1.819 10.090 15.629 2.12e**-**08 *******

treat.L 6.005 1.768 39.250 3.397 0.00157 ******

treat.Q 1.677 1.755 39.210 0.956 0.34509

treat.C 2.006 1.734 39.130 1.157 0.25437

treat**^**4 1.435 1.724 39.090 0.832 0.41047

**---**

Signif. codes**:** 0 ‘*******’ 0.001 ‘******’ 0.01 ‘*****’ 0.05 ‘.’ 0.1 ‘ ’ 1

Correlation of Fixed Effects**:**

**(**Intr**)** tret.L tret.Q tret.C

treat.L 0.016

treat.Q 0.013 0.043

treat.C 0.008 0.026 0.022

treat**^**4 0.003 0.010 0.008 0.005

anova**(**tetmod0, tetmod2**)**

**---------------------------------------------------------------------------------**

Data**:** bouts_data

Models**:**

object**:** sqrt**(**tet**)** **~** **(**1 **|** colony**)**

..1**:** sqrt**(**tet**/**bouts**)** **~** treat **+** **(**1 **|** colony**)**

Df AIC BIC logLik deviance Chisq Chi Df Pr**(>**Chisq**)**

object 3 609.53 615.26 **-**301.76 603.53

..1 7 336.14 349.38 **-**161.07 322.14 281.38 4 **<** 2.2e**-**16 *******

**---**

Signif. codes**:** 0 ‘*******’ 0.001 ‘******’ 0.01 ‘*****’ 0.05 ‘.’ 0.1 ‘ ’ 1
